# Program for the Education and Enrichment of Relational Skills (PEERS^®^) for Italy: A Randomized Controlled Trial of a Social Skills Intervention for Autistic Adolescents

**DOI:** 10.1007/s10803-023-06211-3

**Published:** 2024-01-08

**Authors:** Laura Maria Fatta, Elizabeth A. Laugeson, Dora Bianchi, Italian Peers® team support group, Fiorenzo Laghi, Maria Luisa Scattoni

**Affiliations:** 1 Research Coordination and Support Service, Istituto Superiore di Sanità, Viale Regina Elena 299, 00161 Rome, Italy; 2 Department of Developmental and Social Psychology, Sapienza University of Rome, Via dei Marsi 78, 00185 Rome, Italy; 3 University of California, Los Angeles, Los Angeles, CA USA

**Keywords:** Autism spectrum disorder, Social skills training, Adolescents, International adaptation, PEERS^®^

## Abstract

The *Program for the Education and Enrichment of Relational Skills* (PEERS^®^) is an intervention targeting social skills for autistic adolescents and those with other social challenges. The efficacy of the PEERS^®^ on adolescents has been extensively explored but the program has not been validated in Italy. In the present study, we adapted PEERS^®^ to Italian culture and evaluated its efficacy in an Italian sample. A Randomized Controlled Trial was conducted, analyzing the results of 37 autistic adolescents who were randomly assigned to two groups: experimental group (TG) and waitlist group (WL). The primary outcomes (social abilities) and secondary outcomes (co-occurring conditions, executive functions) were assessed at four time points. No differences were found at pre-treatment between groups on baseline measures and primary outcomes. At post-treatment, significant group differences emerged in primary outcomes (social knowledge and social performance) and secondary outcomes (emotion regulation). The groups’ results pooled together (TG + WL) confirmed the findings at post-treatment and showed further changes in primary outcomes (global social competence and social cognition) and secondary outcomes (externalizing problems, emotive and behavioral total problems, functional problems related to depressive symptoms). The improvements were maintained at a 3-month follow-up, except for global social competence and social cognition. Additionally, new results emerged regarding internalizing problems and global executive functioning. The efficacy of the Italian version of PEERS^®^ was ascertained on primary and secondary outcomes. Innovative findings on emotion regulation, behavioral problems, and depression symptoms also emerged.

*Clinical trial registration information* Program for the Education and Enrichment of Relational Skills (PEERS^®^) for Italy. An RCT’s Study on Social Skills Intervention for Adolescents with Autism Spectrum Disorder (ASD). URL: http://clinicaltrials.gov. TRN: NCT05473104. Release Date: July 21, 2022.

**Supplementary Information:**

The online version contains supplementary material available at 10.1007/s10803-023-06211-3.

## Introduction

Autism Spectrum Disorder (ASD) is a heterogeneous and highly heritable neurodevelopmental disorder defined by two diagnostic criteria: (1) difficulties in reciprocal social communication, and (2) restricted, repetitive patterns of behaviors, interests, or activities (APA, 2013). Social competence is a multidimensional construct including specific verbal and nonverbal behaviors, like asking and answering questions appropriately and giving and acknowledging compliments during the social exchange. Social competencies are operationalized in two clusters: *social knowledge,* related to how to perform prosocial behaviors, and *social performance* which is linked with the difficulties of generalizing social scripts in daily life (Gresham, 1997). Difficulties in social competence may negatively affect relationships with family, peers, and the social environment across the lifespan (Billstedt et al., 2007). Autistic individuals^1^ without intellectual or learning disabilities tend to show poor insight into social contexts and often fail to recognize social rules. They often desire to engage in relationships with others—both autistic and non-autistic people—but frequently fail to find the social resources to make and keep friendships. These difficulties become more evident in adolescence and adulthood by the increase of social demands (Laugeson & Ellingsen, 2014; Moody & Laugeson, 2020), and/or burnout, caused by the effort of masking autistic traits (Higgins et al., 2021). Autistic individuals often cope with social challenges that can be divided in three domains: *social communication*, *social cognition,* and lack of *insight into social cues.* Social communication in autism is characterized by inconsistent patterns, such as hyper-verbosity or being excessively argumentative (Ghaziuddin, 2010). Moreover, the understanding of humor is hampered by the adherence to literal language and misinterpretation of sarcasm and language pragmatics (Winter & Lawrence, 2011). *Social cognition* difficulties influence the Theory of Mind (ToM) and empathy processes, reducing the sharing of affective experiences and limiting social perspective-taking (Baron-Cohen, 1995). These processes are fundamentals to developing reciprocal friendships. Finally, a lack of *insight into social cues* can be a barrier to understanding nonverbal elements of social interaction, such as the use of gestures (Laugeson & Ellingsen, 2014; Moody & Laugeson, 2020). Autistic teens with poor social competencies are highly vulnerable to bullying victimization, ridicule, and bad reputation among their peers (Shtayermman, 2007). Consequently, they are more exposed to peer rejection and/or social avoidance in different contexts, such as school and peers’ social environments (van Roekel et al., 2010). Social withdrawal can lead to low self-esteem, depressive symptoms (Mazurek, 2014), and autistic burnout (Higgins et al., 2021), especially when social difficulties are unrecognized. Therefore, treatments focused on supporting social skills may reduce ASD core difficulties (Gates et al., 2017) and decrease the risk of developing psychiatric co-occurring conditions (Mazurek, 2014).

### Efficacy of Social Skills Training for ASD

The efficacy evaluation of Social Skills Training (SST) for autistic adolescents is currently a relevant research topic that requires further investigation for several reasons. First, social difficulties are the primary cause of challenges in autistic population, regardless of cognitive and verbal abilities, and persist over time (Billstedt et al., 2007). Second, improved social skills are linked to higher self-efficacy and Quality of Life (QoL), which act as protective factors against psychiatric co-occurring conditions (Mazurek, 2014). Lastly, while interventions in childhood are well-tailored (Lai et al., 2014), there is a lack of evidence regarding efficacy for adolescence and adulthood (Ninci et al., 2015). Overall, meta-analyses on SST confirm the efficacy (as internal validity) of SST for ASD, detecting moderate (e.g. Gates et al., 2017) and large (e.g. Wolstencroft et al., 2018) effect sizes. On the contrary, effectiveness (or external validity) is often underreported (Jonsson et al., 2016), although it is a crucial point for clinicians and services (Smith et al., 2007). SST are based on social learning theories and Cognitive Behavior Therapy (CBT) and are predominantly delivered in group-based formats. Among others, one of the most internationally recognized SST for ASD youth is the *Program for the Education and Enrichment of Relational Skills* (PEERS^®^; Laugeson et al., 2009), which is a parent-mediated and manual-based program targeting youth on the autism spectrum.

The program is aimed to teach successful social skills to make and keep friendships, and to handle issues that could lead to peer rejection. Parents are trained to provide social coaching in the natural setting, using prompts and feedback. There are currently two PEERS^®^ curricula for autistic adolescents: one in clinical settings (Laugeson & Frankel, 2010), and the other in school settings (Laugeson, 2014). PEERS^®^ is an innovative model because it suggests ecologically valid social skills, through a Socratic method, and focuses on individual styles and preferences for making and maintaining friends. The program teaching method refers to psychoeducation (or didactic instruction), role-playing demonstrations, perspective-taking questions, cognitive strategies, behavioral rehearsal exercises, performance feedback, assignments, and the review of socialization homework. The lessons focus on the following fourteen social skills: (1) trading information, (2) conversational skills, (3) electronic communication, (4) choosing appropriate friends, (5) appropriate use of humor, (6) peer entry strategies, (7) peer exiting strategies, (8) get-togethers, (9) good sportsmanship, (10) handling teasing, (11) handling bullying, and changing a bad reputation, (12) handling disagreements, (13) handling cyberbulling, rumors and gossip, and (14) graduation (Laugeson & Frankel, 2010). After the first validation study (Laugeson et al., 2009), PEERS^®^ was replicated with a 14-week short-term follow-up (Laugeson et al., 2012), and with a 1–5-year long-term follow-up (Mandelberg et al., 2014). Currently, evidence on the efficacy and effectiveness of PEERS^®^ for Adolescents includes more than 30 studies and one meta-analysis (Zheng et al., 2021). PEERS^®^ has also been adapted in various countries worldwide with minimal changes, yielding similar results to those found in the United States. Initial cultural adaptations were conducted in South Korea (Yoo et al., 2014) and Canada (Marchica & D’Amico, 2016), followed by studies in East Asia (Shum et al., 2019; Yamada et al., 2020) and Israel (Rabin et al., 2018). In Europe, studies have been conducted with Netherlands (Idris et al., 2022) and Polish adaptations (Płatos et al., 2022). Recent evidence shows that PEERS^®^ efficacy in telemedicine is comparable to the in-person version (Adler et al., 2022; Estabillo et al., 2022; Płatos et al., 2022) indicating that the administration methods do not significantly influence treatment effects. This evidence is consistent with findings from other SST models (Soares et al., 2020).

As of now, there is no Italian version of PEERS^®^ and, to the best of our knowledge, no evidence-based SST has been previously implemented in Italy. Therefore, this research represents the first evidence-based SST designed for ASD, which has been translated and adapted to the Italian context.

### Aims of the Current Study

The present study aimed to evaluate the efficacy of the Italian adaptation of PEERS^®^. The program was administered in telemedicine during the COVID-19 lockdown, on a sample of autistic Italian adolescents, using an RCT research design. Specifically, we tested the program efficacy by comparing two groups (experimental and control/waitlist) on primary outcomes, such as social skills, social knowledge, and social performance (RQ1). Moreover, the effects of PEERS^®^ on secondary outcomes, such as co-occurring psychiatric conditions (anxiety, depression, emotional and behavioral issues) and executive functioning, were also evaluated (RQ2). The outcomes were also examined at a 3-month follow-up to verify the maintenance of changes over time (RQ3). Finally, the feasibility and social validity of the PEERS^®^ intervention were investigated (RQ4).

## Methods

### PEERS^®^ Italian Adaptation

The Italian PEERS^®^ version was formulated by the first author and approved by the developer of PEERS^®^ (Dr. Elizabeth Laugeson) before the training implementation. Italian adaptation was created through the synthesis and translations of the manual for adolescents in the clinical setting (Laugeson & Frankel, 2010), the contents changed in the school-based manual (Laugeson, 2014), and the unpublished telemedicine update materials provided during the COVID-19 pandemic by the UCLA PEERS Clinic (Laugeson, personal communication, 2021). Previous PEERS^®^ adaptations in other countries have modified various modules, such as those related to humor and formal aspects of social interaction (Rabin et al., 2018; Shum et al., 2019; Yamada et al., 2020; Yoo et al., 2014) and others have expanded the number of sessions (Płatos et al., 2022; Rabin et al., 2018). Based on these findings, we administered a survey to non-autistic Italian adolescents, to assess the replacement of the content related to adolescent culture in Italy. The survey was completed by 185 over 219 students in a public high school in Rome (84.4%; 67 girls; M = 15.97; SD = 5.02). To evaluate the substitution of appropriate social groups and sources of friends, we asked the youth to indicate which social groups, from the original manual, they were already familiar with and to include any groups not mentioned in the list. Groups that scored over the threshold value ≥ 40% were included. Additionally, to identify adolescents’ activities during get-togethers, we asked them to indicate where and how they spent time with friends. The answers were grouped into labels, and those with at least 15% frequency were included. PEERS^®^ sessions meet weekly and last 90 min over 14 weeks. For the present study, we extended the sessions to 120 min, with a short break in mid-session. This approach differs from other studies, where the sessions were split into 16 separate sessions (Rabin et al., 2018). As indicated by families, the lessons included too much information, so both parents and adolescents required more time to process the contents. Moreover, linguistic factors, speech speed, and the delivery method via telehealth could have influenced the duration of the sessions. To increase fidelity in treatment implementation, additional suggestions for therapists and behavioral coaches have been incorporated, to self-monitor session timing and manage the allocation of reinforcement points. Furthermore, the handouts for parents were detailed to better support their emerging social coach skills. Additionally, handouts for teens were also provided, to support autonomy in managing the materials. Lastly, telemedicine vs. in-person delivery was highlighted to make the manual adaptable for both delivery methods. Differences between the original manual and the Italian adaptation can be found in the S2 Supplementary Materials. In summary, the manual emphasizes the importance of flexible rule application, valuing neurodiversity inclusively, and equips teens with strategies to understand common social scripts. However, non-autistic adolescents should be more encouraged to understand the social cues and needs of autistic people to field accommodation in mutual friendship.

### Study Design

The design was a two-arm RCT study of the PEERS^®^ Italian adaptation. Participants were randomized in the experimental group (Treatment group, TG), attending training immediately, and the control group (Waitlist group, WL), which participated in training after 14 weeks. Each group was divided into two age-based cohorts (12–15 years; 16–18 years). Data were collected at baseline (T0), after 14 weeks (T1: post-intervention for TG and second baseline for WL), after another 14 weeks (T2: follow-up for TG and post-intervention for WL), and finally, over another 14 weeks (T3: follow-up for only WL). The present study was conducted according to CONSORT guidelines (Schulz et al., 2010) (see S1 Supplementary Materials), and the principles of the Declaration of Helsinki or comparable standards. The research and its procedures received ethical approval by Scientific and Ethics Committee of Department of Developmental and Social Psychology, Sapienza, University of Rome (Date: 09.25.2020/No. 871). Informed Consent was obtained from all participants included in the study.

### Recruitment and Screening of Participants

We use the statistical calculation to estimate the sample size. In the original PEERS^®^ study (Laugeson et al., 2009) significant and large effects on primary outcomes were obtained. Therefore, in the present study, we aimed to reach an adequate sample size to detect large effects too (Cohen’s d = 0.80). Given variability in both (TG and WL) groups: σ = 4.86 fixed α = 0.01 and δ = 2.43 with a power 1−β = 0.80 and significance level (*p*-value) of 0.05 (two-tailed), a minimum sample size of 20 participants per group was estimated, to detect significant results with large effect size. Due to the outbreak of the COVID-19 pandemic, participants were recruited online. Invitations to participate were disseminated by national advocacy agencies, stakeholders, and public clinical services supervised by the Italian National Institute of Health. As suggested by the PEERS^®^ developer (Laugeson et al., 2009, 2012), the inclusion criteria for the study were: (a) certified diagnosis of ASD—level 1, (b) fluency in the Italian language (for both adolescents and parents), (c) social difficulties as reported by adolescents and parents, (d) motivation to participate, (e) having not attended other social skills training in the past 12 months, (f) no neurological issues (i.e. epilepsy), no neurosensorial deficit (i.e. visual or auditory), no genetic syndromes (i.e. Fragile X Syndrome, sclerosis tuberose), (g) absence of co-existing major mental illness (i.e. schizophrenia, psychotic disorders, bipolar disorders) or co-occurring severe behavioral problems.

Teen and parent eligibility was screened during individual teleconference interviews using a modified version of the Phone Screening Script and Teen Intake Interview Checklist included in the PEERS^®^ Treatment Manual (Laugeson & Frankel, 2010). The interviews collected information about adolescents (i.e. diagnostic information, social competencies, global functioning), parents (i.e. education and professional level), and family (i.e. live context, geographical area). The Hollingshead Four Factor Index of Socioeconomic Status, which includes parents’ gender, education level, employment, and marital status, allows for the definition of four categories that calculate socioeconomic status as follows: very low SES (0), low SES (10 to 35), medium SES (40 to 65), and high SES (more than 70; Bellina et al., 2020).

Diagnoses were confirmed by clinical staff of the public health services according to the criteria provided in the DSM-5 (APA, 2013), and supported by Autism Diagnostic Observation Schedule-Second Edition (ADOS-2; Lord et al., 2012). After the interview, the clinician who made the diagnosis was contacted to confirm the inclusion criteria and collect further data about the participant’s verbal and total IQ. Verbal abilities were assessed with the Verbal Comprehension Index of Wechsler Intelligence Scale for Children- Fourth Edition (WISC-IV; Orsini et al., 2012; Wechsler, 2004), and IQ level was computed by the Total Score of WISC-IV. Only participants with a verbal comprehension score ≥ 80 and an IQ score ≥ 70 were included in the study.

### Randomization Process

Another staff member who did not attend the recruitment interviews randomly allocated participants to groups (TG vs. WL). Randomization was performed by a parallel-stratified random sequence generation into an online software (http://randomizer.at) with sex and age as the allocation factor. Participants were unidentifiable with sequential numbering. Personal information was concealed allocation by an encrypted database. The clinical team, participants, and parents were blinded to group assignment until informed consent was obtained. Teachers were not informed about their student’s group assignments.

### Italian PEERS^®^ Intervention Procedures

The sessions were conducted according to the PEERS^®^ Italian adaptation, by telehealth between January and July 2021, and were provided for 14 consecutive weeks in 120 min-sessions. Parents and teens participated in parallel sessions in separate online rooms. Each parent received information about how to use the platform, common group rules, and what to expect from the training. Every week, the handouts were sent to parents and adolescents separately, and homework sheets to parents. The sessions started with homework review, for both adolescents and parents, followed by the didactic lesson on a targeted social skill, including appropriate and inappropriate role-play demonstrations and behavioral rehearsal exercises for teens to practice.

The clinical team was composed of five trained treatment leaders and co-leaders, one of whom was a PEERS^®^ Certified Provider who had received comprehensive training and fellowship at the UCLA PEERS^®^ Clinic. The parent and teen-group leaders were licensed clinical psychologists/psychotherapists with at least 7 years of experience in ASD (CBT therapists, Board Certified Behavior Analyst—BCBA^®^, Systemic therapist), and three trainees in clinical psychology served as social coaches. As in other studies (Schohl et al., 2014), the team was trained on PEERS^®^ with intensive 3-day training by the certified provider.

## Measures

### Baseline Measures

Autistic traits in parents were self-evaluated through the Autism Quotient (AQ) (Baron-Cohen et al., 2001; Ruta et al., 2012). Parents also evaluated the daily living skills of adolescents by the Adaptive Behavior Assessment System—Second Edition (ABAS-II) (Ferri et al., 2014; Harrison & Oakland, 2003). Participants’ scores on the baseline characteristics are reported in Table 1.

**Table 1:** Baseline differences in individual characteristics and primary outcomes between groups

Variable		Treatment group (N = 18)			Waitlist group (N = 19)			t(df)/χ^2^	*p*
		M (SD)	Range		M (SD)	Range			
			Min	Max		Min	Max		
Age (years)		15.14 (2.26)	12.3	18.2	15.50 (1.74)	12.2	18.2	− 0.53 (31.98)	.59
Gender (%F)		33.3			26.3			0.64 (1)	.80
IQ	Verbal	116.35 (14.49)	92	138	114.43 (17.01)	86	132	0.34 (29)	.79
	Total^a^	109.65 (10.51)	94	128	106.43 (16.22)	73	133	0.66 (29)	.51
ABAS-II	General	79.67 (15.27)	61	114	73.50 (12.25)	55	100	1.33 (34)	.19
	Conceptual	87.50 (10.30)	70	106	83.22 (10.62)	65	106	1.22 (34)	.23
	Social	76.83 (14.72)	55	106	76.78 (12.74)	58	100	0.01 (34)	.99
	Practical	77.67 (18.31)	50	116	70.56 (16.08)	48	96	1.23 (34)	.22
Autism severity	ADOS-2 Mod.3^b^	9.45 (2.8)	7	14	9.07 (2.75)	5	14	0.34 (23)	.73
Socio-economic status^c^		40.86 (13.96)	16	64	45.63 (11.88)	21	62	− 1.1 (35)	.27
Parental age (years)	Father	55.17 (7.81)	46	73	52.56 (4.97)	38	60	1.2 (34)	.24
	Mother	49.67 (3.01)	43	53	50.11 (5.46)	42	59	− 0.30 (28.30)	.76
AQ	Father	17.76 (6.69)	9	31	17.13 (7.72)	9	33	0.25 (31)	.80
	Mother	12.83 (7.05)	3	24	14.33 (8.48)	4	36	− 0.57 (34)	.56
Primary outcomes									
SRS-P	Total	80.00 (20.98)	47	121	85.67 (18.25)	25	126	− .86 (34)	.39
SRS-T	Total	70.50 (8.56)	53	90	66.11 (9.88)	53	83	1.42 (34)	.16
TASSK-R		16.06 (2.21)	12	21	15.83 (3.69)	9	23	.21 (27.76)	.82

SRS: P (Parents), T (Teachers)

^a^Test available WISC-IV= 31 (4 evaluations above 3 years; 2 use of the other version of Wechsler's scale)

^b^Test available ADOS-2 total score = 25 (6 not reported a quantitative score, 3 have ADOS-G; 2 evaluations above 3 years)

^c^Calculated with Hollingshead Four Factor Index of Socioeconomic Status. The values were grouped into four categories: 0 = very low SES, 10 to 35 = low SES, 40 to 65 = medium SES, and more than 70 = high SES (Bellina et al., 2020)

Due to the COVID-19 pandemic, questionnaires on primary outcomes and secondary outcomes were administered to multi-informant assessors (adolescents, parents, and teachers) on Qualtrics^XM^ and Google Forms platforms. Only teachers were blinded about the group allocation.

### Primary Outcomes

*The Social Responsiveness Scale (SRS)* (Constantino & Gruber, 2005) is a 65-item scale used to assess features of autism spectrum disorder in children and adolescents aged 4 to 18 years, where higher scores indicate more autistic traits. In this study, it served as the primary outcome measure of global social competence, as recommended by systematic reviews (Kasari & Patterson, 2012). The SRS comprises five scales: Social Awareness (SA), Social Cognition (SC), Social Communication (SCo), Social Motivation (SM), Restricted Interests, and Repetitive Behaviors (RIRB). Both parents and teachers completed the questionnaire at each time point, and raw scores were converted into T scores according to the Italian validation, which demonstrated good psychometric proprieties and excellent consistency in previous studies on Italian samples (*α* > .90; Zuddas et al., 2010). In the current study, internal consistency was satisfactory, both for parents (*α* = .89) and for teachers’ version (*α* = .76).

*Quality of Socialization Questionnaire-Revised* (QSQ-R) (Laugeson & Frankel, 2010; Laugeson et al., 2012) is a questionnaire used as a social performance measure that assesses the ability to make and keep friends in natural settings, based on the number of get-togethers. It consists of two dimensions: The Social Initiative Scale, which includes the number of get-togethers hosted by the adolescent, and how many different people who accepted the invitation; the Social Reciprocity Scale, which includes the number of get-togethers the adolescent attended as a guest, and how many different people invited him/her. Therefore, in the present study, we treat these dimensions as separate outcomes, and no composite scale score was reported. Due to this approach and the low number of items, Cronbach’s alpha was not calculated. The age range for this questionnaire is not specified, and there are no normative reference samples. After back-translation and cultural adaptation, the measure was completed separately by parents and adolescents, and raw scores were used to calculate the two scales.

*Test of Adolescent Social Skills Knowledge—Revised* (TASSK-R) (Laugeson & Frankel, 2010; Laugeson et al., 2012) is a 30-item questionnaire used to assess social knowledge where higher scores indicate higher social knowledge. Normative reference sample and cut-off thresholds are unavailable. Previous studies have shown reliability around *α* = .56 in most cases (Laugeson et al., 2009, 2012; Mandelberg et al., 2014; Schohl et al., 2014; Shum et al., 2019). Due to the breadth of the covered domains (two items correspond to each session) and the low correlation between items, this reliability has been considered sufficient (Schohl et al., 2014; Shum et al., 2019). Thus, reliability was not calculated for the present study, in line with other studies (Dolan et al., 2016). For the present study, TASSK-R was completed by adolescents at each time point after back-translation and cultural adaptation, and raw scores were used to calculate a total score.

### Secondary Outcomes

*Child Behavior Checklist (CBCL)* (Achenbach & Rescorla, 2001) is a checklist used to assess developmental psychopathology, evaluates various dimensions, including Syndrome Scales, Internalizing, Externalizing, Total Problems, and DSM-Oriented Scales, with higher scores indicating more severe symptoms. In this study, CBCL was administered in parent, teacher, and youth forms at each time point, with analysis focused on Internalizing, Externalizing, and Total score dimensions. Raw scores were converted into standardized T scores using Italian normative group references that demonstrated excellent consistency for parents (internalizing problems *α* = .83; externalizing problems *α* = .85; total problems *α* = .91), and teachers (internalizing problems *α* = .86; externalizing problems *α* = .92; total problems *α* = .94) (Frigerio et al., 2004). In the current data, internal consistency indicated satisfactory reliability for parents (internalizing problems *α* = .87; externalizing problems *α* = .91; total problems *α* = .95), teachers (internalizing problems *α* = .84; externalizing problems *α* = .88; total problems *α* = .93), and adolescents (internalizing problems *α* = .90; externalizing problems *α* = .78; total problems *α* = .93).

*Multidimensional Anxiety Scale for Children-Second Edition (MASC-2)* (March, 2013) is a 50-item questionnaire used to assess anxiety in children and adolescents aged 8 to 19 years, where higher scores indicate more severe symptoms. It evaluates various dimensions, including Separation Anxiety/Phobias, Generalized Anxiety Disorder (GAD) Index, Social Anxiety, Obsessions and Compulsions, Physical Symptoms, Total Score (indicating the overall severity of anxiety symptoms), and an Anxiety Probability Score (indicating the likelihood of one or more anxiety disorders). Raw scores were converted into standardized T scores according to the Italian validation, where internal consistency, test–retest reliability, and validity (discriminant, convergent, and interrater reliability) demonstrated good psychometric properties (Paloscia et al., 2017). The internal consistency of the MASC total score shows excellent reliability in the current data (*α* = .93). The self-report form was used for the present study and administered to adolescents at each time point.

*Children’s Depression Inventory—Second Edition* (CDI-2) (Kovacs, 2010) is a 28-item questionnaire used to identify depressive symptoms in children and adolescents aged 7 to 17 years, where higher scores indicate more severe symptoms. It provides a Total Score and two dimensions: Emotional Problems and Functional Problems. The raw scores were converted into standardized T scores, using the Italian version, where validity (concurrent, convergent, and discriminating) has been confirmed (Camuffo & Cerutti, 2018). Only the self-report form was used for the present study, administered to adolescents at each time point. The measure demonstrated acceptable reliability in the present sample (total score *α* = .86; Emotional Problems *α* = .81; and Functional Problems* α* = .69), consistent with other Italian studies (*α* = .80; Frigerio et al., 2001).

*Behavior Rating Inventory of Executive Function—Second Edition* (BRIEF-2) (Gioia et al., 2015) assesses executive functioning in children aged 5 to 18 years, where higher standardized T scores indicate more deficits in executive functions. The parent version, used for the present study, consists of 63 items organized into 9 clinical scales, which can be combined to obtain 3 indices: Behavioral Regulation (BRI), Emotional Regulation Index (ERI), and Cognitive Regulation Index (CRI). A Global Executive Composite Score (GEC) can be derived from the sum of the three indices. For the present study, standardized T scores were analyzed using the Italian normative scores. In the Italian validation of the scales, the internal consistency was good to excellent, with reliability scores ranging from .72 to .97 (Marano et al., 2016). The internal consistency obtained in the current data also was excellent (BRI *α* = .83; ERI *α* = .90; CRI *α* = .95; GEC *α* = .96).

### Assessment of the Feasibility

Cultural aspects influence feasibility and interventions should be adapted to the needs of specific cultures. For this reason, feasibility was assessed on the following components: (1) implementation fidelity, (2) social validity (specifically, satisfaction with the intervention), and (3) homework completion and participation.

The use of the manual ensured treatment *fidelity* and the Italian version also provided details on how to handle typical issues in the session, providing additional guidance for therapists. Before each session, a preparatory meeting was held with the PEERS^®^ certified provider and other members of the treatment team to review treatment goals and adaptations discuss clinical issues, and examine monitoring sheets received from parents. After each session, a team briefing was conducted to monitor the application of strategies in behavior rehearsals, the adherence to the protocol during sessions, and the agreement between information delivered by adolescents and parents. The PEERS^®^ certified provider viewed the recordings of 50% of the parent sessions and the supervisor attended 20% of the adolescent sessions to check implementation fidelity.

Following treatment, a satisfaction questionnaire was administered to parents and adolescents to evaluate *social validity* (see S3 Supplementary Materials). The questionnaire investigated the perceived efficacy of the program in changing social skills, the usefulness of each session’s content, the usefulness of the parent sessions (only for parents), and whether they would recommend the program to other families/peers. In addition, participants were asked to indicate three strengths and three weaknesses of the program.

*Homework completion* was evaluated weekly by analyzing the percentage of the homework completed, as indicated in the parents’ sheets, and by considering information provided by both parents and teens during the homework revision.

Finally, social coaches recorded the *participation rate* at each parent and teen session.

### Data Analysis

The Statistical Package for Social Sciences software (IBM^®^ SPSS) version 27.0 was used for statistical analysis. Statistical significance was defined at the conventional level of *p* < 0.05. Independent-sample *t*-tests and Chi-square difference tests were initially performed to evaluate differences between TG and WL groups in the study variables at the baseline (T0).

The treatment efficacy was investigated using a series of repeated measure factorial analyses of variance (ANOVAs), comparing the two groups (TG vs. WL) and the scores obtained in the outcome measures at two time points (T0-T1), through three different models. The Wilcoxon test was used—testing only the first model—for the variables that violated the assumption of normal distribution. In the first model, the group (TG vs. WL) was entered as a between-subjects factor, the time-point (T0 vs. T1) was entered as a within-subjects factor, and each outcome score was entered as a dependent variable. In the second model, the same ANOVAs were repeated including participants’ gender and age as covariates. Finally, in the third model, socioeconomic status, mother and father’s educational level, maternal and paternal age, and both parents’ AQ scores were also added as covariates. When statistically significant Group*Time interaction effects emerged and remained significant in all models, paired-sample *t*-test analyses were run, separately for TG and WL, in order to understand the significance of T0-T1 differences on the dependent variable in each group. Bonferroni’s correction for multiple comparisons was applied, using a critical alpha value of .025 per test (.05/2).

To test additional treatment-related effects and investigate the maintenance of treatment efficacy over time, two repeated measures ANOVAs were performed, considering the overall sample (all participants as a unique group). In the first ANOVA, pre-treatment vs. post-treatment was the within-subjects factor. In the second ANOVA, pre-treatment vs. follow-up was the within-subjects factor. When assumptions of normal distribution were violated for the outcome variables, the ANOVA was substituted with the Wilcoxon test.

The social validity of the PEERS^®^ program was determined by calculating the percentage frequencies of participants’ responses on each dimension. Moreover, the significance of differences between response frequencies was evaluated using a series of Chi^2^ tests. The content of open-ended answers was also qualitatively considered to understand better the quantitative answers.

## Results

**Fig. 1:**
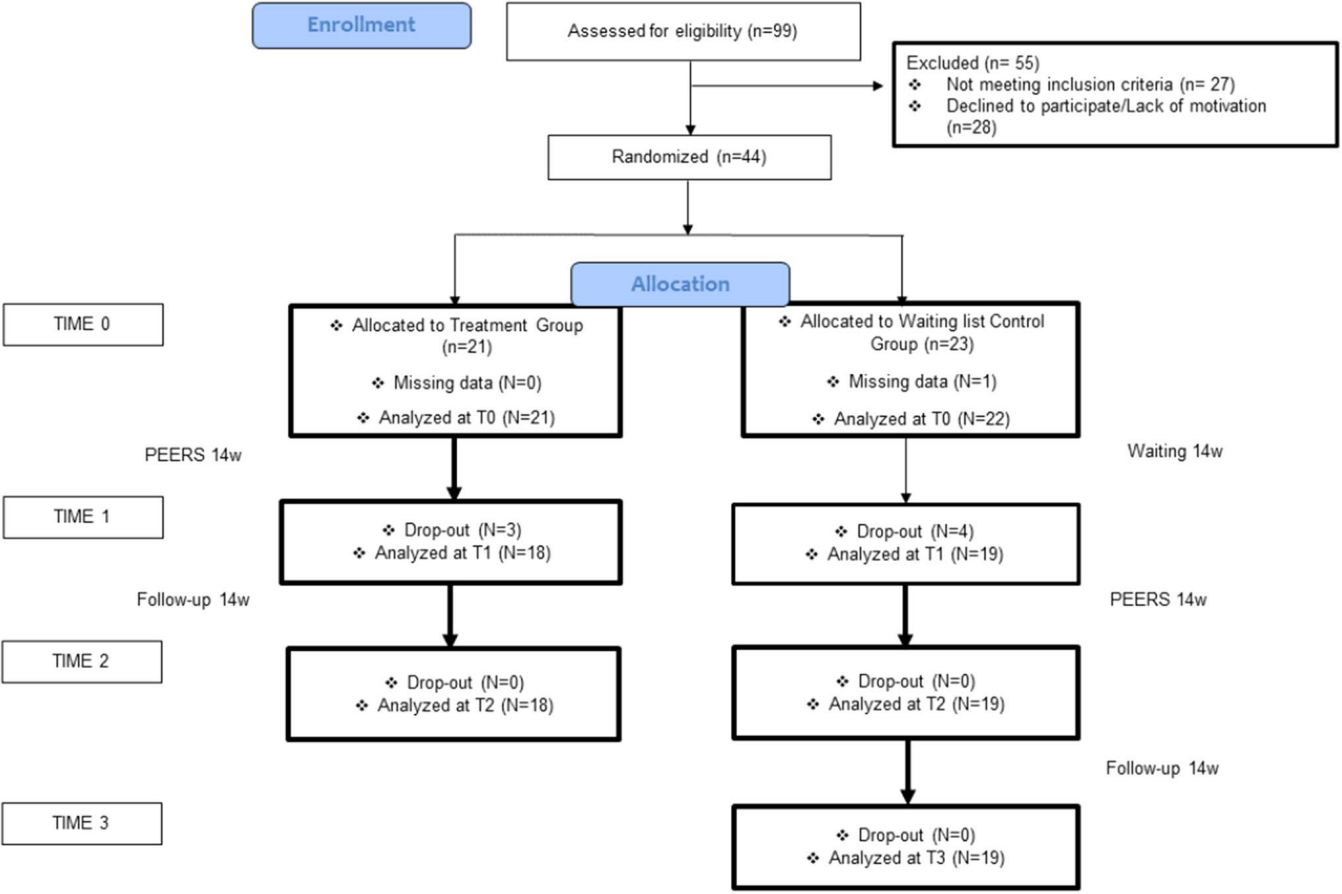
CONSORT flow diagram

The first author conducted ninety-nine teleconference interviews of which fifty-five participants were excluded (twenty-eight adolescents did not meet inclusion criteria, twenty lacked motivation, and the remaining seven refused to participate without providing any reasons). Forty-four participants aged 12–18 years with a diagnosis of ASD (level 1) were enrolled but three in the TG and four in the WL dropped out after randomization and before training started (three for scheduling conflicts, three did not provide information, and one for several behavioral problems). Dropout participants were not replaced, and data were not analyzed. Therefore, the final sample was composed of 37 participants randomly distributed across TG (*n* = 18) and WL (*n* = 19). The process of participant screening, randomization, and research implementation is summarized in Fig. 1.

### Differences Between Groups at the Baseline

Descriptive analyses have been calculated for the overall sample (*M*_age_ = 15.3; *SD*_age_ = 2.0; age range_age_: 12.2–18.2, N_female_ = 11, 29.7%) and cohorts: 12–15 years (TG: *n* = 9; *M*_age_ = 13.1; *SD*_age_ = 0.91; range_age_: 12.3–14.6; WL: *n* = 9; *M*_age_ = 14.0; *SD*_age_ = 1.13; range_age_: 12.2–15.3) and 16–18 years (TG: *n* = 9; *M*_age_ = 17.1; *SD*_age_ = 0.79; range_age_:15.7–18.2; WL: *n* = 10; *M*_age_ = 16.8; *SD*_age_ = . 82; range_age_: 15.10–18.2). Any attrition in outcome measures was registered, except for one participant assigned to the WL whose T0 assessment was unavailable. All participants attended regular public schools.

To assess the differences between the two groups (TG vs. WL) at T0 in categorical variables, Chi^2^ difference tests were conducted. Moreover, no statistically significant differences emerged in the geographical area (*χ*^2^ = .70, *p* = .40; Cramer’s v = .13), where teens were from central (*n* = 17; 45.9%) and northern Italy (*n* = 20; 54.1%). Adolescents lived in urban areas (*n* = 17; 45.9%), towns (*n* = 12; 32.4%), suburbs (*n* = 5; 13.5%) or in rural areas (*n* = 3; 8.1%), and no significant differences were found in terms of live areas (*χ*^2^ = 3.13, *p* = .34; Cramer’s v = .30), sex (*χ*^2^ = .64, *p* = .80; Cramer’s v = .04). For eight participants (21.6%) another family member (across three generations) had an ASD diagnosis, in eight cases (21.6%), at least one parent had autistic traits (broader autism phenotype, BAP), and in one case (2.7%) the adolescent was adopted, and information regarding biological family history was unavailable. No significant differences were found between the groups (TG vs. WL in family history of ASD, *χ*^2^ = 2.77, *p* = .42; Cramer’s v = .27). In the sample, 29.7% had a secondary neurodevelopmental condition (TG vs. WL in neurodevelopmental conditions, *χ*^2^ = .22, *p* = .64; Cramer’s v = .07) and 10.8% of the participants were taking medication for internalizing or externalizing problems (TG vs. WL in drugs, *χ* ^2^ = 1.24, *p* = .26; Cramer’s v = .18) but no significant differences were found between the groups.

Independent-samples *t*-tests were performed entering the groups (TG vs. WL) as the independent variable, and the baseline continuous measures of age, IQ total score, adaptive skills, autism severity, parental age, socioeconomic status, parental autistic traits, and primary outcomes (SRS, TASSK-R), as dependent variables. No statistical differences emerged between the two groups in all examined variables, as shown in Table 1.

### Differences Between Outcomes in the Treatment Group vs. Waitlist Control Group

Treatment effects on primary and secondary outcomes were analyzed by a series of repeated measures factorial ANOVAs, through three different models. The means and standard deviations for the two groups across different time points are summarized in Table 2. The Group*Time interaction effects and the estimated effect sizes are outlined in Table 3. As regards the primary outcomes, a significant interaction effect, with large effect size, was found in TASSK-R, with effects remaining significant also when controlling for all covariates in the second and third models. The subsequent paired-samples *t*-test comparisons indicated that the TASSK-R mean scores were significantly higher at T1 (vs. T0) only for the TG, *t*(17) = − 9.65, *p* < .001, but not for the WL, *t*(17) = − 1.02, *p* = .32. Thus, an improvement in social knowledge was revealed at T1 only in the experimental group.

**Table 2:** Descriptive statistics on primary and secondary outcomes in the two groups across time-points

		Treatment group			Waitlist group			
		(N = 18)			(N = 19)			
		Pre-test (T0)	Post-test (T1)	Follow-up (T2)	Pre-test (T0)	Second Pre-test (T1)	Post-test (T2)	Follow-up (T3)
		M (SD)	M (SD)	M (SD)	M (SD)	M (SD)	M (SD)	M (SD)
Primary outcomes								
SRS-P	Total	80.00 (20.98)	70.11 (14.00)	73.78 (18.92)	85.67 (18.25)	81.74 (15.74)	81.63 (18.44)	80.37 (16.88)
	SA	63.89 (18.41)	59.00 (9.97)	60.67 (12.60)	67.94 (14.87)	66.89 (14.49)	65.21 (13.78)	65.05 (11.27)
	SC	70.94 (18.96)	64.17 (11.83)	63.89 (14.20)	77.22 (18.40)	72.63 (12.21)	69.84 (16.11)	73.84 (13.44)
	SCo	78.17 (17.08)	69.61 (11.82)	72.72 (15.49)	84.33 (17.41)	79.89 (15.60)	80.32 (18.03)	78.11 (17.14)
	SM	73.50 (20.40)	64.89 (16.47)	68.94 (19.54)	77.67 (17.92)	76.16 (16.67)	76.21 (20.66)	75.74 (20.23)
	RIRB	83.89 (23.78)	72.94 (16.73)	76.56 (22.35)	86.72 (21.4)	82.58 (19.30)	83.32 (21.02)	79.21 (17.45)
SRS-T	Total	70.50 (8.56)	69.11 (11.25)	68.06 (12.03)	66.11 (9.88)	65.79 (9.90)	65.89 (10.10)	66.12 (12.51)
	SA	60.00 (9.08)	59.28 (12.75)	57.94 (10.00)	52.94 (9.28)	54.16 (9.47)	55.26 (10.48)	55.88 (12.88)
	SC	65.83 (9.96)	65.39 (11.23)	64.28 (13.07)	63.89 (12.25)	62.37 (11.24)	62.21 (10.39)	64.41 (15.38)
	SCo	71.44 (10.12)	68.61 (10.22)	67.44 (11.57)	65.89 (10.78)	65.42 (9.37)	65.74 (10.35)	65.76 (9.58)
	SM	65.56 (10.08)	63.78 (11.98)	64.44 (12.11)	66.89 (12.97)	66.32 (11.03)	65.74 (8.46)	64.12 (8.51)
	RIRB	72.17 (16.15)	72.61 (17.84)	71.28 (16.89)	65.17 (8.71)	66.53 (13.21)	66.16 (14.85)	65.94 (18.34)
TASSK-R		16.06 (2.2)	25.39 (3.51)	29.94 (2.55)	15.83 (3.69)	16.58 (2.61)	25.21 (2.39)	24.37 (3.91)
Secondary outcomes								
BRIEF-2	GEC	64.22 (13.55)	61.17(12.84)	59.67 (13.12)	64.11 (11.06)	65.42 (10.49)	63.11 (12.40)	60.21 (13.57)
CBCL-P	Internalizing Pr	65.28 (9.36)	62.00 (10.07)	63.17 (8.92)	63.61 (8.37)	57.68 (14.17)	60.89 (8.51)	59.63 (6.99)
	Externalizing Pr	53.44 (10.13)	51.11 (8.31)	51.44 (7.61)	53.11 (7.97)	51.58 (6.82)	49.74 (6.09)	53.00 (6.18)
	Total Pr	61.22 (9.48)	58.44 (8.59)	58.11 (8.08)	61.33 (7.49)	59.16 (8.52)	58.53 (6.63)	57.63 (5.90)
CBCL-T	Internalizing Pr	64.72 (7.20)	63.06 (6.20)	61.89 (7.25)	64.61 (8.96)	63.16 (6.95)	62.47 (6.73)	63.18 (8.92)
	Externalizing Pr	57.11 (6.53)	55.72 (7.28)	53.17 (7.45)	53.61 (6.83)	53.21 (6.26)	54.32 (7.50)	53.82 (7.42)
	Total Pr	59.61 (6.02)	58.28 (6.27)	56.00 (7.80)	58.06 (6.35)	56.95 (6.01)	57.68 (6.69)	58.00 (6.06)
CBCL-Y	Internalizing Pr	61.72 (9.27)	59.78 (11.52)	56.50 (12.52)	60.06 (12.45)	56.79 (13.00)	56.26 (13.60)	52.74 (13.71)
	Externalizing Pr	54.17 (8.88)	51.22 (8.77)	49.33 (8.93)	51.17 (7.61)	50.79 (6.53)	49.05 (7.99)	45.79 (9.81)
	Total Pr	59.94 (8.89)	56.83 (10.68)	53.06 (12.15)	56.89 (10.76)	53.53 (10.77)	52.47 (10.97)	49.16 (13.03)
CDI-2	Total	53.94 (10.46)	52.00 (9.84)	49.56 (10.87)	51.33 (9.60)	50.37 (7.62)	48.95 (7.14)	49.53 (8.90)
MASC-2	Total	59.28 (13.54)	55.61 (12.91)	54.56 (13.33)	57.17 (13.64)	55.53 (13.12)	55.26 (12.42)	54.16 (13.36)

SRS: P (Parents). T (Teachers); *SA* social awareness, *SC* social cognition, *SCo* social communication, *SM* social motivation, *RIRB* restricted interests and repetitive behaviors, *BRIEF-2 GEC* global executive composite, CBCL: P (Parents); T (Teachers); Y (Youth)

**Table 3:** Repeated measures analyses of variance (ANOVA): Pre-Treatment (T0) vs. Post-treatment/Second Pre-test (T1) between groups

		Model 1		Model 2		Model 3	
		F (df)	η^2^_p_	F (df)	η^2^_p_	F (df)	η^2^_p_
Primary outcomes							
SRS-P	Total	2.40 (1,34)	.07	1.99 (1, 32)	.06	1.20 (1, 22)	.05
	SA	0.89 (1,34)	.02	0.62 (1,32)	.02	0.00 (1,22)	.00
	SC	0.37 (1,34)	.01	0.35 (1,32)	.01	0.00 (1,22)	.00
	SCo	1.14 (1,34)	.03	.89 (1,32)	.03	0.36 (1,22)	.00
	SM	2.90 (1,34)	.07	2.50 (1,32)	.07	3.23 (1,22)	.13
	RIRB	3.63 (1,34)	.09	3.24 (1,32)	.09	3.16 (1,22)	.13
SRS-T	Total	0.60 (1,34)	.02	0.53 (1,32)	.02	0.07 (1,22)	.00
	SA	0.78 (1,34)	.02	0.62 (1,32)	.02	0.47 (1,22)	.02
	SC	0.00 (1,34)	.00	0.00 (1,32)	.00	0.00 (1,22)	.00
	SCo	1.71 (1,34)	.05	1.57 (1,32)	.05	0.33 (1,22)	.01
	SM	0.13 (1,34)	.00	0.16 (1,32)	.00	0.11 (1,22)	.00
	RIRB	0.32 (1,34)	.00	0.17 (1,32)	.01	0.04 (1,22)	.00
TASSK-R		51.61 (1,34)***	.60	52.52 (1,32)***	.62	40.79 (1,22)***	.65
Secondary outcomes							
BRIEF-2	GEC	2.10 (1,34)	.06	1.67 (1,32)	.05	0.79 (1,22)	.03
CBCL-P	Internalizing Pr	0.02 (1,34)	.00	0.05 (1,32)	.00	0.12 (1,22)	.00
	Externalizing Pr	0.13 (1,34)	.00	0.20 (1,32)	.01	0.00 (1,22)	.00
	Total Pr	0.08 (1,34)	.00	0.06 (1,32)	.00	0.00 (1,22)	.00
CBCL-T	Internalizing Pr	0.19 (1,34)	.00	0.38 (1,32)	.01	0.04 (1,22)	.00
	Externalizing Pr	0.41 (1,34)	.01	0.55 (1,32)	.02	1.02 (1,22)	.04
	Total Pr	0.18 (1,34)	.00	0.31 (1,32)	.01	0.02 (1,22)	.00
CBCL-Y	Internalizing Pr	0.49 (1,34)	.01	0.67 (1,32)	.02	0.02 (1,22)	.00
	Externalizing Pr	3.17 (1,34)	.08	2.77 (1,32)	.08	2.52 (1,22)	.10
	Total Pr	0.01 (1,34)	.00	0.10 (1,32)	.00	0.61 (1,22)	.03
CDI-2	Total	0.30 (1,34)	.01	0.11 (1,32)	.00	0.01 (1,22)	.00
MASC-2	Total	1.18 (1,34)	.03	0.97 (1,32)	.03	3.80 (1,22)	.15

SRS: P (Parents). T (Teachers); *SA* social awareness, *SC* social cognition, *SCo* social communication, *SM* social motivation, *RIRB* restricted interests and repetitive behaviors, *BRIEF-2 GEC* global executive composite, CBCL: P (Parents); T (Teachers); Y (Youth)

Model 1 = no covariates. Model 2 = Controlled teen’s age, sex, as covariates. Model 3 = Controlled Socioeconomic Status (SES), mother and father’s education, mother and father’s AQ scores, mother and father’s ages, as covariates

*p*-values: ***p < .001; **p < .01; *p < .05. Benchmarks for effect size interpretation correspond to Cohen’s *η*^2^_p_ ≥ .01, Small effect; ≥ .06, Intermediate effect; ≥ .14, Large effect (Cohen, 1988)

With regard to secondary outcomes, no significant interaction effects were found in the main variables reported in Table 3. However, a significant Group*Time interaction effect, with large effect size, was found on the BRIEF-2 Emotion Regulation Index (ERI) measured by parents, *F*(1,22) = 15.20, *p* < .001, *η*^2^_p_ = .41. Regarding the direction of interaction, a significantly lower score emerged at T1 (vs. T0) in TG, *t*(17) = 3.90, *p* = .001 (pre-treatment: *M* = 68.56; *SD* = 15.92; post-treatment: *M* = 59.67; *SD* = 14.54), but not in WL, *t*(17) = − 1.76, *p* = .097 (pre-treatment: *M* = 64.22; *SD* = 11.28; second pre-treatment: *M* = 66.17; *SD* = 13.58), indicating that emotion dysregulation decreased only in the experimental group.

Additionally, the comparison of T0 vs. T1 scores in the QSQ variables, run with the Wilcoxon test, detected a significant increase in the number of get-togethers hosted by participants (QSQ-Social Initiative Scale) only for TG, but not for WL group, in both parent and adolescent versions (*parent version*: T0–T1 differences in TG, z = − 3.412, *p* < .001; T0–T1 differences in WL, z = − .639, *p* = .523; *adolescent version*: T0–T1 differences in TG, z = − 3.086, *p* = .002; T0–T1 differences in WL, z = − .357, *p* = .721). No statistically significant differences emerged across the two time points in the number of invited get-togethers (QSQ- Social Reciprocity Scale), as rated by parents (T0−T1 differences in TG, z = − 1.499, *p* = .134; and T0–T1 differences in WL, z = − 1.022, *p* = .307), or by adolescents (T0-T1 differences in TG, z = − .045, *p* = .964; and T0–T1 differences in WL, z = − .178, *p* = .858).

### Differences Between Pre-treatment, Post-treatment, and Follow-Up in the Overall Sample

Two repeated measures ANOVAs were performed to test additional treatment-related effects and the maintenance of treatment efficacy over time. Table 4 summarizes the means and standard deviations in the overall sample across the time points. The Group*Time interaction effects and the estimated effect sizes are represented in Table 5.

**Table 4:** Descriptive statistics on primary and secondary outcomes in the overall sample (n = 37)

		Pre-treatment	Post-treatment	Follow-up
		M (SD)	M (SD)	M (SD)
Primary outcomes				
SRS-P	Total	80.89 (18.23)	76.03 (17.22)	77.16 (17.96)
	SA	65.43 (16.35)	62.19 (12.32)	62.92 (11.98)
	SC	71.81 (15.65)	67.08 (14.29)	69.00 (14.52)
	SCo	79.05 (16.13)	75.11 (16.06)	75.49 (16.36)
	SM	74.86 (18.37)	70.70 (19.35)	72.43 (19.92)
	RIRB	83.22 (21.30)	78.27 (19.51)	77.92 (19.74)
SRS-T	Total	68.08 (9.45)	67.46 (10.65)	67.11 (12.12)
	SA	57.00 (9.62)	57.22 (11.65)	56.94 (11.36)
	SC	64.05 (10.63)	63.76 (10.78)	64.34 (14.03)
	SCo	68.35 (10.08)	67.14 (10.25)	66.63 (10.53)
	SM	65.95 (10.44)	64.78 (10.22)	64.29 (10.37)
	RIRB	69.27 (14.78)	69.30 (16.47)	68.69 (17.56)
TASSK-R		16.32 (2.40)	25.30 (2.95)	24.65 (3.29)
Secondary outcomes				
BRIEF-2	GEC	64.84 (11.92)	62.16 (12.48)	59.95 (13.17)
CBCL-P	Internalizing Pr	61.38 (12.5)	61.43 (9.18)	61.35 (8.07)
	Externalizing Pr	52.49 (8.52)	50.41 (7.19)	52.24 (6.86)
	Total Pr	60.16 (8.93)	58.49 (7.54)	57.86 (6.95)
CBCL-T	Internalizing Pr	63.92 (7.02)	62.76 (6.40)	62.51 (8.01)
	Externalizing Pr	55.11 (6.61)	55.00 (7.33)	53.49 (7.33)
	Total Pr	58.24 (6.08)	57.97 (6.41)	56.97 (6.98)
CBCL-Y	Internalizing Pr	59.19 (11.46)	57.97 (12.58)	54.57 (13.10)
	Externalizing Pr	52.43 (7.84)	50.11 (8.33)	47.51 (9.43)
	Total Pr	56.65 (10.29)	54.59 (10.91)	51.05 (12.59)
CDI-2	Total	52.11 (9.17)	50.43 (8.58)	49.54 (9.77)
MASC-2	Total	57.35 (13.28)	55.43 (12.48)	54.35 (13.16)

SRS: P (Parents). T (Teachers); *SA* social awareness, *SC* social cognition, *SCo* social communication, *SM* social motivation, *RIRB* restricted interests and repetitive behaviors, *BRIEF-2 GEC* Global Executive Composite, CBCL: P (Parents); T (Teachers); Y (Youth)

**Table 5:** Repeated measures analysis of variance (ANOVA) in the overall sample (n = 37)

		Pre-treatment vs. post-treatment		Pre-treatment vs. follow-up	
		F (df)	η^2^_p_	F (df)	η^2^_p_
Primary outcomes					
SRS-P	Total	4.67 (1,36)*	.11	3.57 (1,36)	.09
	SA	3.17 (1,36)	.08	1.84 (1,36)	.05
	SC	4.22 (1.36)*	.10	2.53 (1,36)	.06
	SCo	3.35 (1,36)	.08	3.55 (1,36)	.09
	SM	3.31 (1,36)	.08	1.39 (1,36)	.04
	RIRB	3.61 (1,36)	.09	3.52 (1,36)	.07
SRS-T	Total	0.48 (1,36)	.01	0.57 (1,36)	.01
	SA	0.02 (1,36)	.00	0.00 (1,36)	.00
	SC	0.07 (1,36)	.00	0.01 (1,36)	.00
	SCo	1.56 (1,36)	.04	1.81 (1,36)	.19
	SM	0.63 (1,36)	.02	0.49 (1,36)	.01
	RIRB	0.00 (1,36)	.00	0.34 (1,36)	.01
TASSK-R		258.43 (1,36)***	.88	133.74 (1,36)***	.78
Secondary outcomes					
BRIEF-2	GEC	2.72 (1,36)	.07	12.05 (1,36)***	.25
CBCL-P	Internalizing Pr	0.00 (1,36)	.00	0.00 (1,36)	.00
	Externalizing Pr	5.28 (1,36)*	.13	0.04 (1,36)	.00
	Total Pr	2.28 (1,36)	.06	4.40 (1,36)	.11
CBCL-T	Internalizing Pr	0.77 (1,36)	.02	0.96 (1,36)	.03
	Externalizing Pr	0.00 (1,36)	.00	1.25 (1,36)	.03
	Total Pr	0.05 (1,36)	.00	1.18 (1,36)	.03
CBCL-Y	Internalizing Pr	1.74 (1,36)	.05	11.84 (1,36)***	.25
	Externalizing Pr	8.97 (1,36)**	.20	14.75 (1,36)***	.29
	Total Pr	5.99 (1,36)*	.14	19.06 (1,36)***	.35
CDI-2	Total	2.66 (1,36)	.07	3.71 (1,36)	.09
MASC-2	Total	2.92 (1,36)	.07	3.49 (1,36)	.08

SRS: P (Parents). T (Teachers); *SA* social awareness, *SC* social cognition, *SCo* social communication, *SM* social motivation, *RIRB* restricted interests and repetitive behaviors, *BRIEF-2 GEC* global executive composite, CBCL: P (Parents); T (Teachers); Y (Youth)

*p*-values: ***p < .001; **p < .01; *p < .05. Benchmarks for effect size interpretation correspond to Cohen’s *η*^2^_p_ ≥ .01, Small effect; ≥ .06, Intermediate effect; ≥ .14, Large effect (Cohen, 1988)

As regards the primary outcomes, the ANOVAs showed significant differences from pre-test to post-test in SRS-Total and SRS-Social Cognition scores (only parent version), with medium effect sizes (see Table 5), indicating that autistic social traits significantly decreased immediately after treatment according to parent reports (see mean scores in Table 4). However, the significance of these differences was not maintained in the comparison between pre-test and follow-up time points. Significant differences between pre-and post-treatment, with large effect sizes, also emerged in adolescent social skills knowledge on the TASSK-R, which also held significance in the comparison between pre-test and follow-up (see Table 5). Specifically, the TASSK-R scores were significantly higher in the post-intervention, and this improvement was maintained at 3 months following treatment (see Table 4). The differences of get-togethers hosted by participants (QSQ-Social Initiative Scale), analyzed with the Wilcoxon test, confirmed the significant differences between pre-and post-intervention, as reported both by adolescents, z = − 2.452, *p* = .014 (*M*_Pre_ = 3.49, *SD*_Pre_ = 5.26; *M*_Post_ = 6.00, *SD*_Post_ = 4.22; *M*_Follow-up_ = 4.84, *SD*_Follow-up_ = 4.65), and by parents, z = .045, *p* < .001 (*M*_Pre_ = 3.19, *SD*_Pre_ = 5.54; *M*_Post_ = 8.35, *SD*_Post_ = 6.16; *M*_Follow-up_ = 7.08, *SD*_Follow-up_ = 6,61), indicating that the amount of social encounters hosted by adolescents increased after treatment. When comparing pre-treatment and follow-up, the difference remained significant in the parents’ assessment, z = − 3.522, *p* < .001, but not in the adolescents’ version, z = − 1.640, *p* = .101. No significant differences were found in the number of get-togethers to which participants were invited (QSQ-Social Reciprocity), as reported by parents, in pre-and post-treatment comparison, z = − 1.380, *p* = .168, and in the pre-treatment and follow-up comparison, z = − 4.14, *p* = .679 (*M*_Pre_ = 3.49, *SD*_Pre_ = 5.26; *M*_Post_ = 6.00, *SD*_Post_ = 4.22; *M*_Follow-up_ = 4.84, *SD*_Follow-up_ = 4.65). In the adolescent version, QSQ-Social Reciprocity scores showed no significant differences between pre-and post-treatment, z = − 2.452, *p* = .647, but a significant increase was showed between pre-treatment and the 3-month follow-up, z = − 2.027, *p* = .043 (*M*_Pre_ = 2.92, *SD*_Pre_ = 5.22; *M*_Post_ = 3.22, *SD*_Post_ = 4.79; *M*_Follow-up_ = 4.14, *SD*_Follow-up_ = 5.52), indicating that improvements in this variable may be detectable in the long-term.

Regarding secondary outcomes, significant differences emerged between pre-and post-treatment on the CBCL externalizing problems, reported by parents, with moderate effects size, and CBCL externalizing and total problems, reported by adolescents, with large effects sizes, in both dimensions. However, only the results reported by adolescents in CBCL externalizing and total problems were confirmed between pre-test and follow-up, while the parent-reported differences were no more significant at follow-up. Specifically, externalizing behaviors and total problems significantly decreased immediately after treatment and, only in the adolescents’ perception, this amelioration was maintained after 3 months. Moreover, a significant difference, with large effect size, also emerged between pre-treatment and follow-up in the CBCL internalizing problems as reported by adolescents, indicating that internalizing symptoms were significantly reduced some months after treatment (see Table 4 and 5). In addition to what is reported in Table 5, there was a significant difference, with large effect size, between pre-treatment and post-treatment, on the parent-reported BRIEF-2 Emotion Regulation Index (ERI), *F*(1,36) = 16.53, *p* < .001, η^2^_p_ = .31, indicating a significant reduction of emotion dysregulation from pre-treatment (*M* = 67.22; *SD* = 14.45) to post-treatment (*M* = 60.54; *SD* = 14.45). The difference remained significant also when comparing BRIEF-2 ERI scores between pre-treatment and follow-up, *F*(1,36) = 18.63, *p* < .001, η^2^_p_ = .34. Moreover, only when comparing pre-treatment with follow-up, a significant difference with large effect size also emerged in the Global Executive Composite Score (GEC) of the BRIEF-2 (see Tables 4, 5). Thus, emotion dysregulation was reduced immediately after treatment and this improvement was maintained over time. Global deficits in executive functions instead were reduced in the long-term, with significant decrement detectable only 3 months after treatment. With regard to the psychopathological questionnaires completed by adolescents, there was a significant difference with large effect size between pre-and post-treatment in the CDI-2 Functional Problems subscale, *F*(1,36) = 7.50, *p* = .010, η^2^_p_ = .17 indicating that interpersonal problems related to depressive symptoms were significantly reduced from pre-treatment (*M* = 57.46; *SD* = 9.69) to post-treatment (*M* = 54.54; *SD* = 10.52). Moreover, this difference remained statistically significant between pre-treatment and follow-up, *F*(1,36) = 11.72, *p* = .002, η^2^_p_ = .25.

### Social Validity and Adherence to Treatment

To assess *acceptability*, we collected data on the perceptions of both parents and adolescents regarding the impact of the training on social skills. We calculated the percentage frequencies for each answer to analyze the data. Full results are available in the S3 Supplementary Materials. However, 68% of adolescents rated PEERS^®^ as a very helpful intervention, and 62% would recommend the program to other peers. Regarding the strengths of the program, the adolescents frequently mentioned two key benefits. Firstly, they appreciated the opportunity to meet other peers with similar autistic profiles. Secondly, they found the availability of a set of specific rules in complex social situations to be highly beneficial. They pointed out that in the past they thought they understood the strategies covered in the training, but they did not know when and how to enact them. After the training, they reported feeling more confident in their social abilities, had enhanced their understanding of how to interpret the thoughts and emotions of others, and felt ready “to get out of their comfort zone.” However, one participant referred to experiencing anxiety and stress during the training. Overall, adolescents reported that they enjoyed the structured activities and games but found the didactic lessons boring and challenging at times.

Following training, 89% of parents reported an improvement in their child’s social competencies, 84% evaluated very helpful the teen group (homogeneous in autistic characteristics) and 92% would recommend PEERS^®^ to other families. According to the parent report, particular strengths of the program were the interaction with other families with similar concerns, the use of concrete strategies explained in steps, the monitoring and supervision of homework assignments, the therapists’ abilities to individualize the treatment, the dissemination of handouts, and the role-playing demonstrations. However, parents provided contrasting responses on the length of the sessions (too long vs. too short) and on the management of the session (too much time on homework review vs. reduced time to unstructured discussion of their experiences—not provided in the program).

The results of parents’ and adolescents’ *satisfaction rates* for each session, evaluated using Chi^2^ tests, show that statistically significant differences between groups were observed only in session 3 (Electronic communication, *χ*^2^(4) = 13.32, *p* = .10; Cramer’s V = .42). Specifically, teens reported lower engagement by more frequently endorsing “not helpful” for session 3, as compared to parents (8.1% teens vs. 2.7% parents), indicating that session 3 was more helpful for parents compared to their children.

With regard to adherence to treatment, the mean and percentage of the *participation rates* and *homework completion* were calculated by assigning values 1 to attendance, 0 to absence, 0.5 if the parent was late by more than 30 min, and 0.5 if adolescents were late or turned off their camera. The participation rate was 94.88% among adolescents, and 94.98% among parents. The rate of homework completion by adolescents was 79.95%. In summary, these findings promote the acceptability of the intervention from both parent and teen reports.

## Discussion

PEERS^®^ is a structured international program that has been recently validated in different European countries (Idris et al., 2022; Płatos et al., 2022), as well as in telehealth or hybrid delivery (Adler et al., 2022; Estabillo et al., 2022; Płatos et al., 2022). To the best of our knowledge, this study is the first evidence of an Italian adaptation of manualized SST and the first of the PEERS^®^ program. The study aimed to evaluate the efficacy of PEERS^®^ in an Italian autistic adolescent sample. Specifically, the two main aims were to verify whether the adolescents participating in the training (TG) presented significant changes in primary outcomes (i.e. social skills, social knowledge, and social performance; RQ1) and in secondary outcomes (i.e. co-occurring psychiatric conditions and executive functioning; RQ2), in comparison with the waitlist control group (WL). Moreover, we aimed to verify if the expected improvements were maintained over time after a 3-month follow-up (RQ3) and to evaluate the social validity and feasibility of the intervention (RQ4).

With regard to RQ1, results on primary outcomes confirm the efficacy of the Italian adaptation of PEERS^®^ in both adolescent and parent reports. Social knowledge (measured with TASSK-R) significantly improved after training, even when the model was tested with covariates and a lower critical alpha level. This result is consistent with most adaptations of PEERS^®^ in different countries using different covariates (Yoo et al., 2014) or without covariates (Płatos et al., 2022; Shum et al., 2019; Yamada et al., 2020). A limitation in previous SST studies was the lack of information on the subsequent generalization of learned social skills (Gates et al., 2017). While understanding social labels is necessary, it is not sufficient for the systematic generalization of social behaviors across various life contexts (Kasari & Patterson, 2012). In the present study, the effects of the training on social performance are highlighted in the increase of get-togethers with peers hosted by participants (QSQ-R, Social Initiative Scale), as reported by parents and adolescents. This pattern was also observed in other studies (Laugeson et al., 2012; Marchica & D’Amico, 2016), while some studies reported effects from only one informant (Laugeson et al., 2009; Rabin et al., 2018; Schohl et al., 2014; Yoo et al., 2014). In general, comparing the results across studies is challenging because some studies examined hosted (social initiative) and invited get-togethers (social reciprocity) as a total score (social engagement), rather than discrete dimensions (e.g. Mandelberg et al., 2014; Yamada et al., 2020). In the current study, the QSQ-R scale was also not normally distributed, similar to other studies (Schiltz et al., 2018; Yamada et al., 2020). As a result, multivariate modeling was not conducted to control for other potentially relevant covariates (e.g. baseline anxiety levels).

Regarding secondary outcomes (RQ2), to our knowledge, this is the first PEERS^®^ study to employ a comprehensive neuropsychological assessment of executive functions. Previous studies had measured executive functions using a NEPSY-II subtest but yielded no significant results (Lordo et al., 2017). However, our findings revealed a significant reduction in emotional dysregulation scores (BRIEF-2 Emotional Regulation Index) for the TG. Therefore, as a secondary effect, the intervention demonstrated an improvement in emotional regulation skills, despite it not being one of the primary treatment goals.

Consistent with previous PEERS^®^ studies (Rabin et al., 2018; Yamada et al., 2020; Yoo et al., 2014) we analyzed the overall sample to detect additional treatment effects. Combining TG and WL outcome scores, new findings emerged in global social competence, indicated by significant reductions in autistic traits measured by the SRS Social Cognition subscale and the SRS Total Score. These results are aligned with other PEERS^®^ evidence (e.g. Laugeson et al., 2012). In the overall sample, significant treatment effects were observed also in secondary outcomes. Notably, improvements were identified in externalizing problems (CBCL parents and adolescents reports), as well as, in total problems (CBCL adolescent reports), indicating a general amelioration of behavioral problems after treatment. Similar findings in total problems were also reported by Yamada et al. (2020), while Yoo et al. (2014) found improvements only in internalizing problems. Additionally, interpersonal problems related to depressive symptoms (CDI-2 Functional Problems scale) were significantly reduced after treatment. This subscale assesses functional problems arising from depressive symptomatology that may affect interpersonal relationships. Our findings indicated an amelioration of depressive symptoms after the participation of PEERS^®^ groups, positively influencing peers, school, and family relationships, consistent with prior evidence (Schiltz et al., 2018; Yoo et al., 2014). Moreover, the present study expands the existing literature by providing the first evidence of the specific influence of depressive symptoms on interpersonal relationships. Externalizing behaviors and depressive symptoms are common in ASD youth and represent critical stressors for adolescents and their families (Mazurek, 2014). Therefore, the treatment effects on these secondary outcomes have noteworthy implications for clinicians and service providers, in their support for autistic adolescents with co-occurring psychiatric conditions.

Regarding the maintenance of treatment gains over time (RQ3), significant findings from pre-treatment and follow-up comparisons were still present three months after treatment. With regard to primary outcomes, social knowledge scores (TASSK-R) remained significantly higher at follow-up (vs. baseline), indicating a sustained improvement by the training, and the impact of parents acting as social coaches. Improvements in the social initiative scale (QSQ-R by parents) were also maintained at follow-up, suggesting that adolescents continued to initiate social gatherings with their peers. Additionally, adolescents reported an increase in the social reciprocity scale (QSQ-R) at the follow-up, indicating more frequent invitations to get-togethers from peers compared to pre-treatment. This finding, consistent with other cultural adaptations (Yoo et al., 2014), is likely a result of the gradual development of social reciprocity and repeated peer interaction.

With regard to the maintenance of secondary outcomes over time, the improvement in emotion regulation skills (BRIEF- 2 ERI scale) was confirmed at the follow-up. Moreover, a new result emerged, showing a significant reduction in global deficits in executive functions (BRIEF-2 GEC scale). This constitutes the first evidence of PEERS^®^ demonstrating positive effects on global executive functioning, indicating a promising research direction. Future studies should be directed to understand how the PEERS^®^ intervention might influence changes in global executive functioning. Regarding behavioral problems, not only reductions in total and externalizing problems were observed, but adolescent reports also revealed significantly reduced internalizing problems three months following treatment. These findings provide new and relevant insights into the treatment effects in emotional and behavioral problems, complementing previous studies (Yamada et al., 2020; Yoo et al., 2014). The improvement in depressive symptoms (CDI-2 subscale Functional Problems) was also maintained at follow-up. This finding is particularly noteworthy as this is the first study to confirm the effect of PEERS^®^ training on depressive symptoms over time since previous PEERS^®^ studies have not included follow-up assessments of depressive symptoms (Hong et al., 2019; Schiltz et al., 2018).

Regarding feasibility (RQ4), the study showed excellent results with a 100% treatment completion rate, 95% participation adherence, and 80% homework completion. The use of a manualized procedure, team supervision, video review by independent practitioners, and post-session meetings ensured treatment fidelity. For future replications, a specific checklist could be adopted to quantitatively monitor implementation quality. The findings on social validity (RQ4) confirmed the intervention’s ecological value (Laugeson & Ellingsen, 2014; Moody & Laugeson, 2020). Both adolescents and parents considered the training useful, with 62% of adolescents and 92% of parents indicating they would recommend it to others. Notably, parents perceived session 3 (Electronic communication) as more helpful, while adolescents struggled to fully engage with it. This session focused on appropriate social rules in electronic contexts, such as exchanging contact information, starting and ending calls with peers, and being careful with communication on social networks. This discrepancy may be due to the fact that autistic adolescents without cognitive impairment, despite difficulties in relationship rules on social media, are often tech-savvy and report understanding cyberbullying risks (e.g. they knew who a troll was, what "spamming" meant, etc.). Consequently, the skills taught in the session might have been familiar to them, leading to less engagement. In contrast, parents appreciated the contents and observed their children using skills they had rarely or never seen in the past.

In conclusion, the Italian adaptation of PEERS^®^ was found to be effective in improving social skills in both components: behaviors in relational situations with peers (social knowledge) and efficacy experienced in practicing these social behaviors in their real-life context (social performance). The program also showed positive secondary effects on emotional dysregulation, depressive symptoms, and behavioral challenges. Following the intervention, the positive effects persisted over time for both primary and secondary outcomes. Furthermore, new domains appear to improve in the long-term (over 3 months after treatment), specifically internalizing problems and global executive functioning. Finally, PEERS^®^ was shown to be well accepted by parents and adolescents in the Italian context and perceived as helpful in dealing with peer relationships.

## Limitations and Future Directions

Despite the relevance and novelty of these results, this study also has some limitations. First, the results should be interpreted with caution due to the potential bias introduced by participants or their parents overestimating perceived social skills (Wolstencroft et al., 2018). Additionally, the lack of statistical significance in teacher evaluations might indicate a possible bias (blinding bias). The pattern of non-significance in blind assessor evaluations is reported in other PEERS^®^ studies (e.g. Laugeson et al., 2009, 2012; Shum et al., 2019). However, in this study, certain contributing factors might have negatively influenced the results, as teachers completed the assessment at each time points were different, and the introduction of social distancing and remote learning during the COVID-19 pandemic limited the opportunities for teachers to observe students’social behaviors at school. Consequently, teachers’ reports of social functioning may not represent a valid assessment in the context of this study. Second, due to COVID-19 lockdowns, it was not possible to include direct observation measures despite this practice being suggested by systematic reviews (Mirzaei et al., 2020) and often used in other international PEERS^®^ adaptations (Idris et al., 2022; Rabin et al., 2018). Future research should also consider including observational tests administered by other staff members outside the treatment setting. Despite classmates potentially having more opportunities than teachers to observe the social behaviors of autistic adolescents, currently, there are no studies that include them in the assessment process. Third, the effects of PEERS^®^ on parental and family outcomes have not been evaluated, thus it is unknown how the intervention influenced familial well-being. While parental stress has been studied in previous PEERS^®^ research (Corona et al., 2019; Karst et al., 2015), changes to quality of life have yet to be explored. Fourth, the homework completion was not differentiated by behavioral assignments: socio-conversational tasks (e.g. in-group and non-group member phone calls) vs. socio-relational tasks (e.g. get-togethers), as previous studies did (Shum et al., 2019; Yoo et al., 2014). For teens, arranging get-togethers could be more challenging and not everyone was willing to meet online during the COVID-19 pandemic, as emerged in the families’ reports. Therefore, the task completion rate, in our study, might be higher for tasks involving conversation and lower for social activities due to external factors. Fifth, in this study, the participants were not undergoing other SST, but some of them were receiving other psychological treatments. In future studies, it would be interesting to verify whether PEERS^®^, in combination with psychotherapy, could have more relevant effects on secondary outcomes. Six, fidelity was ascertained with wide methods, but future studies might include a checklist to monitor the implementation. Regarding methodological issues, the research evidence does not support differences in delivery methods. However, it may be beneficial to encourage additional RCT studies with in-person training to further validate the efficacy of PEERS^®^ in the Italian context. Noteworthy, a crucial research perspective is the evaluation of the effectiveness of the PEERS^®^ program through community studies. A community-based study would also complete the intervention validation process (Smith et al., 2007), which in the PEERS^®^ literature was currently tested only through a study with a reduced sample size (Hill et al., 2017). Performing an effectiveness study might be challenging but is particularly important, as it is critically imperative for clinicians to have access to "real-world" evidence-based interventions and could provide new information for clinical services and health economics, still poorly evaluated in studies of SST.

## Supplementary Information

Below is the link to the electronic supplementary material.

Supplementary file1 S1 File. CONSORT checklist (PDF 260 KB)

Supplementary file2 S2 File. Italian adaptation details (PDF 745 KB)

Supplementary file3 S3 File. Social validity and adherence to treatment details (PDF 644 KB)

## Footnotes

1 There is an ongoing debate regarding the most respectful expression for identifying autistic people, specifically: the person-first language “person with ASD” is considered offensive, “person on the spectrum” stigmatizing, while the identity-first language “autistic person” is preferred (Botha et al., 2021). Thus, in the present study, we adopted the identity-first language to identify the target of intervention and the level of support.
