# Supplementary material for: Program for the Education and Enrichment of Relational Skills (PEERS®) for Italy: A Randomized Controlled Trial of a Social Skills Intervention for Autistic Adolescents
Source: J Autism Dev Disord. 2024 Jan 8;55(1):202–20. doi: 10.1007/s10803-023-06211-3 (PMC11802708; doi:10.1007/s10803-023-06211-3)
Supplement: Supplementary file 2 — Supplementary file2 S2 File. Italian adaptation details (PDF 745 KB) [file 10803_2023_6211_MOESM2_ESM.pdf]

***Program for the Education and Enrichment of Relational Skills (PEERS®) for Italy: A Randomized Controlled Trial of a Social Skills Intervention for Autistic Adolescents***

Journal of Autism and Developmental Disorders

**Authors:**

Fatta Laura Maria[1,2], Laugeson Elizabeth A[3], Bianchi Dora[2], Italian Peers® team support group[†], Laghi Fiorenzo[2*], Scattoni Maria Luisa[1].

**Affiliations:**

[1]Research Coordination and Support Service, Istituto Superiore di Sanità, Viale Regina Elena 299, 00161 Rome, Italy; laura.fatta@iss.it (L.M.F. ID ORCID: 0000-0001-6451-8077); marialuisa.scattoni@iss.it (M.L.S.).

[2]Department of Developmental and Social Psychology, Sapienza University of Rome, Via dei Marsi 78, 00185 Rome, Italy; dora.bianchi@uniroma1.it (D.B.); fiorenzo.laghi@uniroma1.it (F.L.)

[3]University of California, Los Angeles, CA, USA; elaugeson@mednet.ucla.edu (E.A.L.)

[†]Italian Peers® team support group includes (in alphabetical order): Antei, A., Carnovale, C., Giammello, F., Iannucci, I., Melis, A.

**Corresponding Author**:

Fiorenzo Laghi, Ph.D, Psy.D,

Department of Developmental and Social Psychology,

Sapienza University of Rome,

Via dei Marsi 78, 00185 Rome

E-mail: fiorenzo.laghi@uniroma1.it

The general changes introduced in the Italian version are:

- *Golden rules.* Fundamental rules for each session were identified through a graphic symbol (a star at the apex of the rule) to stress the focus on the session's target.
- *Role-playing videos.* The role-playing videos were transcribed and adapted into Italian by three translators, following a blind procedure. To solve the inconsistencies that emerged among translators, agreement by two translators was required, while the third was consulted if necessary. Finally, the original role-play videos were subtitled.
- *Telemedicine adaptations.* The homework assignments and behavioral rehearsals were modified to be flexibly and applicable for both in-person and for remote delivery. During the COVID-19 lockdown, teens were prevented from practicing with external peers, thus we chose to keep socialization homework assignments within the PEERS® group and additional behavioral rehearsals. These modifications allowed everyone to practice without the pressure of failing to perform the required tasks.
- *Participant checkout.* The PEERS® model provides a short participant checkout meeting at the end of each session. In the Italian version, this checkout was expanded from 10 to 15-20 minutes, to individualize the treatment, as well as negotiate homework completion between the adolescent and parents. Members of the treatment team suggested specific strategies to best suit the teen, give individual specialized support, sustain motivation, and strengthen the generalization of skills.
- *Lesson Handouts.* The original version of PEERS® for adolescents only includes handouts for parents. In the Italian version, according to the PEERS® for Young Adults manual (Laugeson, 2017), handouts were also provided for teens, to support autonomy in managing the materials.

Reference: Laugeson, E. A. (2017). *PEERS for young adults: social skills training for adults with autism spectrum disorder and other social challenges*. New York, NY: Routledge.

Details about differences between the original manual and the Italian adaptation can be found in Table 1.

*Table 1. PEERS® Italian adaptations for each session.*

| Session | *Didactic Lesson* | *Theme* | *Adaptation* | *Rationale* |
|---|---|---|---|---|
| 1 | Trading information | Characteristics of good friendship | (A+P). Add two new features: *Sharing experiences* and *not always taking yourself too seriously* | Emerged as important characteristics of friendships during the training. |
| | | Session rules | (A+P). *Don't brag and be a little more serious when you're first getting to know someone* | Moved from 2nd session by recommendation of the program developer. |
| | | Jeopardy categories | (A). Modified "school spirit" | Unfamiliar term for Italian students, and similar to other adaptations (Yamada et al., 2020). |
| 2 | Conversational skills | Common conversational topics among teenagers | (A+P). Deleted "social clubs" and "applying to college"; Inserted: fashion blogger | Inconsistent with Italian youth culture, similar to other adaptations (Shum et al., 2019). |
| | | Source of friends - Activities | (P). The introduction of common teen activities from session 4 was moved to session 2. | The brainstorming about the link of teen interests with the context where they might meet friends was moved to support parents in developing coach strategies before the specific homework assignment, consistent with other adaptations (Yamada et al., 2020). |
| 3 | Electronic communication | Electronic forms of communication. | (A) Inserted WhatsApp and deleted My Space. | Outdated forms of electronic communication were eliminated, and newer forms were added, consistent with other adaptations (Rabin et al., 2018; Yamada et al., 2020; Yoo et al., 2014). |

| Session | *Didactic Lesson* | *Theme* | *Adaptation* | *Rationale* |
|---|---|---|---|---|
| | | Rules for starting and ending phone calls | (A+P). Two rules for starting a phone call are *Ask for the person you are calling by name* and *Say who you are*. | Outdated rules relating to phone usage were replaced with updated rules regarding mobile phones, including when/how to use them. |
| | | Rules for starting a phone call | (A+P). Inserted a shorter version for starting phone calls, deleting. the step *Ask for the person you are calling by name* and *Say who are you*. Inserted the step *Say hello and ask how he/she is*. | Some steps have been deleted because in most cases adolescents do not use them in informal contexts. |
| | | Rules for ending a phone call | (A+P). One step is to *Tell the person it was nice talking with him or her* has been changed to *Tell the person (if it was so) it was nice talking with him or her (this rule is not mandatory; it's nice to share if you felt it).* | Saying was nice talking with them is not always well accepted by ASD teens who perceive themselves as forced to do it. This point was further clarified add to say it if you felt it. |
| | | Rule: leaving a voicemail | (A+P). The rule has been replaced by *leaving a voice message*. | Voicemail is not used among young people, rather it is important to know how to manage a voice message correctly. |
| | | Source of friends - Activities | (P). Culturally irrelevant activities were deleted and typical social activities among Italian teens were included. Deleted: e.g. yearbook, science club. Added: online video chess course, post-production picture programs, and apps. Modified: going to car fairs (added vintage or not); going to a car showroom (added better with adults); photography club was replaced with photography tour groups. | Cultural adaptations were based on Italian survey results. Similar changes have been done in previous adaptations (Rabin et al., 2018; Shum et al., 2019; Yamada et al., 2020; Yoo et al., 2014). The focus is on activities and the discussion about social groups was postponed to session 4. Parents were overwhelmed contemplating both aspects together, consequently, the sources of friends homework assignment was moved to session 4. |
| 4 | Choosing appropriate friends | Source of friends -Social groups | (A+P). New groups were added: fashion bloggers, youtubers, soccer fans, trapper fans, League of Legends geeks, and Fortnite geeks, while other groups were deleted (e.g. cheerleaders and surfers). | The inclusion or exclusion of social groups resulted from the survey among Italian adolescents, similar to other adaptation studies (Shum et al., 2019; Yamada et al., 2020; Yoo et al., 2014). |
| 5 | Appropriate use of humor | Behavioral rehearsal | (A). Modified the joke in the behavioral rehearsal. Added behavioral rehearsal for *giving a courtesy laugh*. | Consistent with other adaptations (Rabin et al., 2018; Shum et al., 2019; Yamada et al., 2020; Yoo et al., 2014), the joke for paying attention to humor feedback was replaced to be culturally relevant. While *laughing at you* and *laughing with you* can be intuitive, giving a *courtesy laugh* indicates that the joke was not adequate or funny, which can be confusing for autistic youth. Consequently, a behavioral rehearsal for *giving a courtesy laugh* was added. |
| 6 | Peer entry strategies | Appropriate and inappropriate times and places | (A+P). Appropriate/inappropriate times were separated from appropriate/inappropriate places to avoid confusion. An additional inappropriate time was included: when a teacher explains something in class. Additional appropriate places were included: the dining hall, the park, the school, the playroom, and cultural associations. Additional | To avoid confusion, times and places to start conversations were separated. Times and places have been modified according to routine and cultural context. |

| Session | *Didactic Lesson* | *Theme* | *Adaptation* | *Rationale* |
|---|---|---|---|---|
| | | for starting a conversation | inappropriate places were included: the church, on the bus, in a shop, lined up outside the shop, in an elevator. | |
| 7 | Peer exiting strategies | No changes | | |
| 8 | Good sportsmanship | Session order reversed with session 9 | (A+P). The order of sessions for Good Sportsmanship and Get-togethers was reversed, with the former preceding the latter. | To be consistent with recent changes to the original PEERS®, the order of sessions 8 and 9 was reversed by recommendation of the program developer. |
| 9 | Get-togethers | Session order reversed with session 8<br>Activities to do together | (A+P). Common get-together activities have been categorized into classes: mealtime activities, sports activities, paid outdoor activities, unpaid outdoor activities, and indoor activities. Activities such as having a barbecue, playing mini-golf, and baseball were eliminated and replaced with more culturally appropriate activities such as playing football, going to a shopping center, and having a takeaway pizza. | Culturally inappropriate social activities in Italy have been eliminated and replaced with culturally appropriate activities, which were identified from the adolescent survey, similar to other adaptations (Rabin et al., 2018; Yamada et al., 2020; Yoo et al., 2014). |
| | | Steps for beginning a get-together | (A+P). One of the steps for beginning a get-together is to "*Say hello to your guest,*" which might include a hug or some type of physical greeting. This greeting was modified to respect social distancing during COVID-19. | This adaptation was required because of COVID-19 social distancing guidelines when it was not possible to have close contact. |
| | | Steps for beginning the get-together. | (A+P). We modified some steps including which rooms to show when showing your friend around your home, and how to answer when your friends turns down your offer for something to drink or eat. | When showing a friend around during a get-together in Italy, it is usual to limit the rooms to those that a teenager may need (e.g. the bathroom, the kitchen, and the personal bedroom). Other adaptations have modified this step to be culturally appropriate as well (Rabin et al., 2018; Yamada et al., 2020; Yoo et al., 2014).<br>A guest might turn down an offer of food or drink at first to be polite. Instead, putting some drinks on a table would be more culturally appropriate in Italy. |
| | | Steps for beginning the get-together. | (A+P). Modified steps for online get-togethers have been added. For example, rather than show the rooms of the house (that are not relevant), if it is the first online meeting, it is possible to show something from one's bedroom.<br>Uncommon online platforms were deleted (e.g. Google Hangouts, and Facetime). | Online get-togethers rarely include a host and a guest, so adjustments to rules were necessary. |
| | Handling teasing | No changes | | |

| Session | *Didactic Lesson* | *Theme* | *Adaptation* | *Rationale* |
|---|---|---|---|---|
| 11 | Handling bullying, and bad reputation | Rules for managing physical bullying | (A+P). One rule for handling physical bullying is to *avoid the bully*. The original rule suggests not walking near the bully's locker. This suggestion has been modified with another example: *If during a break the bully is in a specific corridor, should we walk in the same direction?*<br><br>Another example has been deleted about what to do if someone has a weapon with him at school. | Necessary adaptations to Italian school contexts. |
| | | Bad Reputation | (A+P). Attention has been focused on the reasons why somebody can have a bad reputation, giving importance to changing one's appearance. The rules were changed by shifting the focus to problem behaviors (including the wrong use of humor and communication). The original rules were *Lay Low, Follow the Crowd, Change your look, Own up to your previous reputation, and Find a source of friends with common interests.* Rules 2,3,4 have been replaced by *Do not draw attention to yourself inappropriately, re-evaluate your appearance to change your reputation and become aware of your previous reputation*. | In Italian culture, although aesthetics plays an important role, it is not common to cope with problems related to one's reputation by making aesthetic shocking changes (such as changing hair color or style) or by admitting to having a bad reputation to peers.<br><br>In addition, we included an introduction to explain the rules by which the therapist promotes behaviors according to individual needs and values. |
| 12 | Handling disagreements | Behavioral rehearsal | (A+P). Other examples for the behavioral rehearsal have been added *(you feel misunderstood because we don't agree on a certain topic; you feel upset because I like things that you do not.)* | New examples were introduced to support ASD teens in feeling more comfortable with peers, consistent with other adaptations (Yoo et al., 2014). |
| | | Modified step | (A+P). One step for handling disagreements is to *Repeat what they said.* This step has been modified with *Repeat what the other seems to feel*. | The purpose of the strategy is to give an emotional reflection and show empathy. Repeating what the other person says was misunderstood by Italian teens as teasing; so was reworded to make it clearer. |
| | | Modified step | (A+P). The step to *Say you're sorry* was replaced with *Say you're sorry about how the other person feels.* | Saying sorry about the content of the discussion is not always well accepted by ASD teens who may be more concerned with being right. Since the focus is not on being wrong or right, but on the emotional response, the step was reworded to be clearer, also consistent with other adaptations (Yamada et al., 2020; Yoo et al., 2014). |
| 13 | Handling cyberbullying, rumors and gossip | Modified rule | (A+P). *Spread the rumor about yourself* has been changed to *Spread a NEW rumor about yourself.*<br><br>It has been also clarified that the NEW rumor must not be invented. | The strategy for handling rumors and gossip is complicated and is based on several steps which aim is to make people forget the current gossip, and spread a new rumor that the person who is the victim of the gossip is surprised that someone would believe the rumor. The reaction of surprise should make people think that the original gossip was wrong or unimportant. This point was further clarified in the adaptation with the modification of describing this strategy as a "NEW" rumor. |

| Session | *Didactic Lesson* | *Theme* | *Adaptation* | *Rationale* |
|---|---|---|---|---|
| 14 | Graduation | Rules | (A). Parent and adolescent sessions have become more similar. The strategies to support the adolescents once the program is over are reviewed with both parents and teens. | The graduation party was thought to be very long and ceremonial, with the formal awarding of certificates. It was preferred to include a game (for the adolescents) with a summary of the sessions and to work toward short and long-term goals. |

The original monitoring sheet – filled out by parents - provided three open-ended questions. In the Italian version, the sheets were drafted as a checklist, so that parents could flag the acquisition of the sub-components of the teen's skill learned for each rule step, and self-monitor their social coaching. Comments and additional examples were included to help the clinician understand the progress of homework assignments and the generalization of the skills.

*Monitoring Sheet SESSION 1: TRADING INFORMATION*

| | | |
|---|---|---|
| **HOMEWORK SHEET**<br>Please fill out this worksheet *before* the next meeting and bring it with you (if the course is live) or return it via email the day before the next session. Answer each question and write additional comments about the assignments in the space provided. | | |
| ***Teen's Name:*** | | |
| ***Who fills in*** | ☐ mother | ☐ father |
| ***Trade information (with parents):*** | | |
| -Did you review the rules with your teen before practicing? | YES<br>Commentary: | NO<br>Commentary: |
| -Did you support your child and reinforce them when they performed the skill? | YES<br>Commentary: | NO<br>Commentary: |
| -Did your child find a common interest with you or another family member? | YES<br>Commentary: | NO<br>Commentary: |

| | | |
|---|---|---|
| -After identifying common interests, did you ask, "What could we do to share that interest if we were hanging out?" | YES<br>Commentary: | NO<br>Commentary: |
| -Did you provide social coaching (i.e. did you support them through all these steps by providing suggestions or prompts and feedback)? | YES<br>Commentary: | NO<br>Commentary: |
| ***In-group phone calls or video chats (CALLER role):*** | | |
| <u>Before phone call/video chat</u><br>- Was your son/daughter available to make the phone call on the scheduled day and time? | YES<br>Commentary: | NO<br>Commentary: |
| Did you review the rules with your teen before making the phone call/video chat? | YES<br>Commentary: | NO<br>Commentary: |
| <u>During</u> phone call/video chat<br>-Did your son/daughter have traded information with the other teen? | YES<br>Commentary: | NO<br>Commentary: |
| -Did your son/daughter find a common interest with the other teen? | YES<br>Commentary: | NO<br>Commentary: |
| <u>At the end of the</u> phone call/video chat -Did your son/daughter discuss the phone call/video chat with you? | YES<br>Commentary: | NO<br>Commentary: |
| -Did you support your teen and compliment them when they reported to you that they had performed the skill? | YES<br>Commentary: | NO<br>Commentary: |
| - After identifying common interests, you asked, "What could you do with your friend, to share that interest, if you hang out with them?" | YES<br>Commentary: | NO<br>Commentary: |
| -Did the phone call/video chat last 5 to 20 minutes? | YES<br>Commentary: | NO<br>Commentary: |
| - Did you social coach (i.e. did you support them through all these steps by providing suggestions or prompts and feedback)? | YES<br>Commentary: | NO<br>Commentary: |
| ***In-group phone calls or video chats (RECEIVER role):*** | | |

| | | |
|---|---|---|
| Before phone call/video chat<br>- Was your son/daughter available to make the phone call or video chat on the scheduled day and time? | YES<br>Commentary: | NO<br>Commentary: |
| Did you review the rules with your teen before making the phone call/video chat? | YES<br>Commentary: | NO<br>Commentary: |
| During phone call/video chat<br>-Did your son/daughter trade information with the other teen? | YES<br>Commentary: | NO<br>Commentary: |
| -Did your son/daughter find a common interest with the other teen? | YES<br>Commentary: | NO<br>Commentary: |
| At the end of the phone call/video chat<br>-Did your son/daughter discuss the phone call/video chat with you? | YES<br>Commentary: | NO<br>Commentary: |
| -Did you support your teen and compliment them when they reported to you that they had performed the skill? | YES<br>Commentary: | NO<br>Commentary: |
| - After identifying common interests, you asked, "What could you do with your friend, to share that interest, if you hang out with him/her?" | YES<br>Commentary: | NO<br>Commentary: |
| -Did the phone call/video chat last 5 to 20 minutes? | YES<br>Commentary: | NO<br>Commentary: |
| - Did you social coach (i.e., did you support them through all these steps by providing suggestions or prompts and feedback)? | YES<br>Commentary: | NO<br>Commentary: |
