# Supplementary material for: Program for the Education and Enrichment of Relational Skills (PEERS®) for Italy: A Randomized Controlled Trial of a Social Skills Intervention for Autistic Adolescents
Source: J Autism Dev Disord. 2024 Jan 8;55(1):202–20. doi: 10.1007/s10803-023-06211-3 (PMC11802708; doi:10.1007/s10803-023-06211-3)
Supplement: Supplementary file 3 — Supplementary file3 S3 File. Social validity and adherence to treatment details (PDF 644 KB) [file 10803_2023_6211_MOESM3_ESM.pdf]

Program for the Education and Enrichment of Relational Skills (PEERS®) for Italy: A Randomized Controlled Trial of a Social Skills Intervention for Autistic Adolescents

Journal of Autism and Developmental Disorders

Authors:

Fatta Laura Maria[1,2], Laugeson Elizabeth A[3], Bianchi Dora[2], Italian Peers® team support group[†], Laghi Fiorenzo[2*], Scattoni Maria Luisa[1].

Affiliations:

[1]Research Coordination and Support Service, Istituto Superiore di Sanità, Viale Regina Elena 299, 00161 Rome, Italy; laura.fatta@iss.it (L.M.F. ID ORCID: 0000-0001-6451-8077); marialuisa.scattoni@iss.it (M.L.S.).

[2]Department of Developmental and Social Psychology, Sapienza University of Rome, Via dei Marsi 78, 00185 Rome, Italy; dora.bianchi@uniroma1.it (D.B.); fiorenzo.laghi@uniroma1.it (F.L.)

[3]University of California, Los Angeles, CA, USA; elaugeson@mednet.ucla.edu (E.A.L.)

[†]Italian Peers® team support group includes (in alphabetical order): Antei, A., Carnovale, C., Giammello, F., Iannucci, I., Melis, A.

Corresponding Author:

Fiorenzo Laghi, Ph.D, Psy.D,

Department of Developmental and Social Psychology,

Sapienza University of Rome,

Via dei Marsi 78, 00185 Rome

E-mail: fiorenzo.laghi@uniroma1.it

Table 1. Acceptability: parents' perceptions of the training's impact (frequencies %).

	Worse	*Same*	*Improved*
How would you judge your teen's social skills before the PEERS program compared to current skills?	3	8	89
	Not helpful	*a little helpful*	*Very helpful*
For your teen, this intervention was:	0	24	76
The sessions for parents were:	0	32	68
Overall, how would you evaluate the PEERS group that your teen attended?	0	16	84
	No	*Maybe*	*Definitely*
Would you recommend participation in the PEERS program to other families?	0	8	92

Table 2. Acceptability: adolescents' perceptions of the training's impact (frequencies %).

	Not helpful	*a little helpful*	*Very helpful*
How do you judge the PEERS as a whole?	7	27	68
	No	*A little*	*Very*
Do you think the PEERS group has helped you improve your social skills?	8	49	41
Did attending the PEERS program feel you more confident in social situations?	11	49	38
Did attending the PEERS program feel you less anxious in social situations?	24	41	30
Did you enjoy participating in the PEERS program?	8	35	54
	No	*Maybe*	*Definitely*
Would you recommend participation in the PEERS program to a friend?	8	27	62
Do you feel that you have changed since you participated in the PEERS program?	16	43	38

Table 3. Satisfaction rate of each session of PEERS®

N. session	Didactic Lesson	Adolescents (%)			Parents (%)			$x^2(4)$
		Very helpful	*a little helpful*	*Not helpful*	*Very helpful*	*a little helpful*	*Not helpful*	
1	Trading information	75.7	18.9	5.4	81.1	18.9	0.0	2.07
2	Conversational skills	62.2	32.4	5.4	75.7	21.6	2.7	3.34
3	Electronic communication	40.5	51.4	8.1	62.2	35.1	2.7	13.32*
4	Choosing appropriate friends	35.1	48.6	16.2	54.1	40.5	5.4	3.32
5	Appropriate use of humor	51.4	37.8	10.8	48.6	48.6	2.7	2.83
6	Peer entry strategies	67.6	27.0	5.4	75.7	24.3	0.0	2.99
7	Peer exit strategies	59.5	37.8	2.7	67.6	32.4	0.0	3.01
8	Good sportsmanship	35.1	56.8	8.1	59.5	29.7	10.8	2.60
9	Get-togethers	67.6	24.3	8.1	83.8	16.2	0.0	.82
10	Handling teasing	56.8	35.1	8.1	78.4	18.9	2.7	1.30
11	Handling bullying and bad reputation	40.5	45.9	13.5	54.1	43.2	2.7	2.04
12	Handling disagreements	62.2	29.7	8.1	78.4	21.6	0.0	5.51
13	Handling cyberbullying, rumors and gossip	59.5	35.1	5.4	56.8	29.7	13.5	3.98

p-values: *** p < .001; ** p < .01; * p < .05.

Table 4. Participation rates and homework completion

	Treatment Group (n=18)				*Waiting list Group (n=19)*				*Total (n=37)*			
	M(sd)	*%*	*range*		*M(sd)*	*%*	*range*		*M(sd)*	*%*	*range*	
Adolescents:			min	max			min	max			min	max
Participation rates (max.=14)	13.36(.92)	95.44	11	14	13.21(1.54)	94.36	9	14	13.28(1.26)	94.88	9	14
Homework completion (max=48)	38.22(4.72)	79.63	24	43	38.53(6.96)	80.26	18	47	38.38(5.90)	79.95	18	47
Parents:												
Participation rates (max.=14)	13.69(.42)	97.82	13	14	12.92(1.34)	92.29	9	14	13.30(1.06)	94.98	9	14

Social validity and adherence to treatment

The questionnaire was adapted from another PEERS® study involving participants with Turner Syndrome.

Reference: Wolstencroft, J., Mandy, W., & Skuse, D. (2018). Protocol: New approaches to managing the social deficits of Turner Syndrome using the PEERS program. *F1000Res*, *29*(7), 1864. https://doi.org/10.12688/f1000research.15489.2

Satisfaction questionnaires

Satisfaction survey, parent version

Please sign the appropriate answer

**1. How much did this session help you?**	**useless**	**quite useful**	**very useful**
Trading information	□	□	□
Conversational skills	□	□	□
Electronic communication	□	□	□
Choosing appropriate friends	□	□	□
Appropriate use of humor	□	□	□
Peer entry strategies	□	□	□
Peer exit strategies	□	□	□
Get-togethers	□	□	□
Good sportsmanship	□	□	□
Handling teasing	□	□	□
Handling bullying and bad reputation	□	□	□
Handling disagreements	□	□	□
Handling cyberbullying, rumors and gossip	□	□	□

2. How would you judge your teens' social skills before PEERS compared to his/her current skills?

□ Worse □ Same □ Better

Please tell us more:

……………………………………………………………………………………………

……………………………………………………………………………………………

……………………………………………………………………………………………

3. For your son/daughter this training was:

□ useless □ quite useful □ Very useful

Please, tell us what you have found useful or not:

……………………………………………………………………………………………

……………………………………………………………………………………………

…………………………………………………………………………………………

4. The sessions for parents were:

□ useless □ quite useful □ very useful

Please tell us what you found useful or not:

…………………………………………………………………………………………

…………………………………………………………………………………………

…………………………………………………………………………………………

5. Overall, how would you judge the PEERS group that your son/daughter attended?

□ useless □ quite useful □ very useful

Please tell us more:

…………………………………………………………………………………………

…………………………………………………………………………………………

…………………………………………………………………………………………

6. Please tell us what you think about the room organization/online logistic:

…………………………………………………………………………………………

…………………………………………………………………………………………

…………………………………………………………………………………………

7. In your opinion, is there anything we should have done differently?

…………………………………………………………………………………………

…………………………………………………………………………………………

…………………………………………………………………………………………

8. Would you recommend the PEERS program to other families?

□ No □ Maybe □ Certainly

Please tell us more:

…………………………………………………………………………………………

…………………………………………………………………………………………

…………………………………………………………………………………………

9. Could you tell us three strengths and three weaknesses of the PEERS program?

<u>Strengths</u>

1.……………………………………………………………………………………

2.……………………………………………………………………………………

3.………………………………………………………………………………………

Weaknesses

1.………………………………………………………………………………………

2.………………………………………………………………………………………

3.………………………………………………………………………………………

Thank you for your answers!

Satisfaction survey, adolescent version

Please sign the appropriate answer

1. How much did this session help you?	useless	quite useful	very useful
Trading information	□	□	□
Conversational skills	□	□	□
Electronic communication	□	□	□
Choosing appropriate friends	□	□	□
Appropriate use of humor	□	□	□
Peer entry strategies	□	□	□
Peer exit strategies	□	□	□
Get-togethers	□	□	□
Good sportsmanship	□	□	□
Handling teasing	□	□	□
Handling bullying, and bad reputation	□	□	□
Handling disagreements	□	□	□
Handling cyberbullying, rumors and gossip	□	□	□

2. Overall, how do you consider the PEERS?
□ useless □ quite useful □ very useful
Please let us know more about your experience:

……
……
………………………………………………………………………

3. Do you think that the PEERS helped you to improve your social skills?
□ No □ a little □ a lot
Please tell us more:
………………………………………………………………………………………………
………………………………………………………………………………………………
………………………………………………………………………………………………

4. Do you think attending the PEERS program makes you feel more confident in social situations?
□ No □ a little □ a lot
Please tell us more:
………………………………………………………………………………………………
………………………………………………………………………………………………
………………………………………………………………………………………………

5. Do you think attending the PEERS program makes you feel less anxious in social situations?
□ No □ a little □ a lot
Please tell us more:
………………………………………………………………………………………………
………………………………………………………………………………………………
………………………………………………………………………………………………

6. Please tell us what you think about the room organization/online logistic:

……………………………………………………………………………………………
……………………………………………………………………………………………
……………………………………………………………………………………………

7. Did you enjoy attending the PEERS?
□ No □ a little □ a lot
Please tell us more:
……………………………………………………………………………………………
……………………………………………………………………………………………
……………………………………………………………………………………………

8. Would you recommend the PEERS program to a friend?
□ No □ Maybe □ Certainly

Please tell us more
……………………………………………………………………………………………
……
……………………………………………………………………………………

9.? Do you feel you have changed since you attended the PEERS program?
□ No □ Maybe □ Certainly

Please tell us more
……………………………………………………………………………………………
……
……………………………………………………………………………………

10. Other comments:
……………………………………………………………………………………………
……………………………………………………………………………………………

Thank you for your answers!
